# Machine Learning-Based Frailty Prediction and Classification in Community-Dwelling Older Adults: A Systematic Review of Validation, Explainability, and Implementation Readiness

**DOI:** 10.3390/healthcare14111543

**Published:** 2026-06-01

**Authors:** Seungmi Kim, Myung-Jun Shin, Byung Kwan Choi, Zoran Obradovic, Daniel J. Rubin, Jong-Hwan Park

**Affiliations:** 1 Department of Convergence Medicine, Pusan National University School of Medicine, Yangsan 50612, Republic of Korea; delos1758@gmail.com; 2 Biomedical Research Institute, Pusan National University Hospital, Busan 49241, Republic of Korea; drshinmj@gmail.com (M.-J.S.); spine@pusan.ac.kr (B.K.C.); 3 Department of Rehabilitation Medicine, Pusan National University School of Medicine, Yangsan 50612, Republic of Korea; 4 Department of Rehabilitation Medicine, Pusan National University Yangsan Hospital, Yangsan 50612, Republic of Korea; 5 Department of Neurosurgery, Pusan National University School of Medicine, Yangsan 50612, Republic of Korea; 6 Department of Neurosurgery, Pusan National University Hospital, Busan 49241, Republic of Korea; 7 Center for Data Analytics and Biomedical Informatics, Temple University, Philadelphia, PA 19122, USA; zoran.obradovic@temple.edu; 8 Department of Computer and Information Sciences, Temple University, Philadelphia, PA 19122, USA; 9 Lewis Katz School of Medicine, Temple University, Philadelphia, PA 19140, USA; daniel.rubin@tuhs.temple.edu; 10 Department of Clinical Bio-Convergence, Graduate School of Convergence in Biomedical Science, Pusan National University School of Medicine, Yangsan 50612, Republic of Korea; 11 Convergence Medical Institute of Technology, Pusan National University Hospital, Busan 49241, Republic of Korea

**Keywords:** frailty, machine learning, prediction model, explainable artificial intelligence, public health

## Abstract

**Highlights:**

**What are the main findings?**

- ML-based frailty models show task-dependent discrimination: classification of current frailty status (n = 7) yielded AUROCs of 0.70–0.98 (top performers: CatBoost at 0.97–0.98 and logistic regression at 0.96–0.97 under same-cohort temporal validation), whereas incident prediction (n = 6) yielded internal AUROCs of 0.70–0.81 and external (same-cohort temporal) AUROCs of 0.58–0.85, and a single trajectory-prediction study reported an internal AUROC of 0.70 without external validation, indicating that the field has not yet achieved consistent generalization in prognostic tasks.
- Key predictors consistently include age, functional status (ADL/IADL), multimorbidity, cognitive function, and social or behavioral factors, reflecting the multidimensional nature of frailty.

**What are the implications of the main findings?**

- Current ML-based frailty models remain at an early translational stage (Technology Readiness Level 4–6); under PROBAST, only 2 of 14 studies (14.3%) met both low risk of bias and low applicability concern, and no study documented evidence for the Implementation or Maintenance domains of RE-AIM, indicating that prospective implementation evaluation is required before routine real-world use.
- Integration with public health systems requires explainability, low-burden inputs, and governance frameworks addressing fairness, usability, and workflow integration.

**Abstract:**

**Background and Objectives:** Frailty is a multidimensional vulnerability in older adults; the Fried phenotype and Frailty Index are clinically informative but labor-intensive, limiting scalability for community screening. Machine learning (ML) can model heterogeneous, high-dimensional data, but real-world adoption is constrained by heterogeneity in definitions, predictors, validation strategies, and explainability. We systematically synthesized ML-based studies of frailty prediction and classification in community-dwelling older adults, examining validation rigor, explainability, and implementation readiness. **Methods:** This systematic review followed PRISMA 2020 and was registered in PROSPERO (CRD420251081555). PubMed, Embase, Web of Science, and Scopus were searched on 4 July 2025, with a supplementary IEEE Xplore and ACM Digital Library search conducted on 12 May 2026. Eligible studies included community-dwelling adults aged ≥60 years, ML-based frailty prediction or classification, sample ≥ 1000, and publication in a peer-reviewed journal indexed in the Web of Science Core Collection; hospital-based studies were excluded. Risk of bias and reporting quality were assessed with PROBAST (Prediction Model Risk of Bias Assessment Tool) and TRIPOD (Transparent Reporting of a Multivariable Prediction Model for Individual Prognosis or Diagnosis); implementation readiness was assessed with the RE-AIM (Reach, Effectiveness, Adoption, Implementation, Maintenance) framework and a Technology Readiness Level (TRL)-style rubric. Findings were synthesized narratively. **Results:** Fourteen studies (development cohorts 1230–86,133 participants) were included; the supplementary IEEE/ACM search identified 42 records but yielded no additional eligible studies. Classification of current frailty status (n = 7) yielded AUROCs (area under the receiver operating characteristic curve) of 0.70–0.98, with the highest values likely reflecting partial label overlap with frailty components; incident prediction (n = 6) yielded internal AUROCs of 0.70–0.81 and same-cohort temporal AUROCs of 0.58–0.85; independent external validation was uncommon. Only 2 of 14 studies had both low overall risk of bias and low applicability concern (PROBAST); the field is concentrated at TRL 4–6, with no study at TRL 7 or higher and none documenting Implementation or Maintenance domains of RE-AIM. **Conclusions:** ML-based frailty models show heterogeneous discrimination and limited readiness for routine community use. Priorities include standardized task-type-specific definitions, independent external validation, calibration and decision-curve reporting, transparent predictor disclosure, and prospective implementation evaluation.

## 1. Introduction

Health is increasingly understood not merely as the absence of disease, but as a continuum defined by the maintenance of functional capacity and resilience [1,2,3]. In later life, subtle declines in intrinsic capacity—across physical, cognitive, emotional, and social domains—can precipitate adverse outcomes such as falls, hospitalization, disability, and mortality [1,2,3,4,5]. Identifying these declines early and intervening in a timely manner are central goals of older adult care [2,5]. From a public health perspective, these goals necessitate large-scale risk stratification in primary care and community settings, where resources are limited and decisions must be made at the population level [1,2,6].

However, conventional frailty assessment tools require multi-item clinical, functional, and performance-based measurements, often involving trained personnel, equipment, and standardized environments [7,8,9]. As a result, their measurement burden constrains scalability, limiting real-time screening and routine deployment in community-based practice [1,4,6]. Machine learning (ML) has emerged as a potential solution by enabling automated risk stratification from heterogeneous, routinely collected data, yet its translation into public health practice remains incomplete [10,11,12].

Importantly, ML-based frailty studies encompass two conceptually distinct tasks that are often conflated: classification (diagnostic identification of current frailty status) and prediction (prognostic estimation of incident frailty or progression) [13,14,15]. Classification models frequently rely on contemporaneous predictors that overlap with frailty definitions themselves, raising concerns about circularity and inflated performance estimates [7,13]. In contrast, prediction models aim to forecast future frailty risk and are more directly aligned with prevention and resource planning [1,5,15]. When studies do not clearly distinguish these objectives, interpretation and comparison across studies, as well as assessment of real-world utility, become challenging [13,14,16].

ML methods are structurally well suited to frailty modeling, given their capacity to capture nonlinearity, high-dimensional interactions, and cumulative risk patterns across heterogeneous data sources spanning electronic health records, survey and claims data, and sensor-derived measurements [10,11,12]. Although ML can, in principle, enable screening with relatively low-burden inputs [1,6,11], practical feasibility varies substantially with predictor measurement burden, and many proposed models remain difficult to operationalize at scale [1,6]. Moreover, existing ML-based frailty models are limited by heterogeneity in frailty definitions, predictors, algorithms, performance metrics, and validation strategies; insufficient cross-institutional external validation; incomplete evaluation of calibration and equity; and limited explainability of internal decision mechanisms [11,13,14,16,17,18,19]. These gaps undermine trust, interpretability, and operational readiness in clinical and public health settings [1,12,20].

Accordingly, model evaluation should extend beyond discrimination metrics (e.g., AUROC [area under the receiver operating characteristic curve], AUPRC [area under the precision–recall curve]) to include calibration, decision curve analysis, and the policy implications of threshold selection [15,16,19,21]. Explainable artificial intelligence (XAI) is therefore essential for translation [12,20,22]. Global explainability clarifies which risk factors drive predictions at the population level, informing policy priorities, while local explainability elucidates why specific individuals are classified as high risk, supporting individualized intervention planning [20,22,23]. Implementation is further strengthened when explainability is paired with practical delivery mechanisms—such as web calculators, nomograms, or risk scores—workflow integration (e.g., embedding within EHR [electronic health record] systems), and post-deployment monitoring with ongoing recalibration [10,14,16,18,19]. Whether such deployment-related dimensions are met in practice is the central question of implementation science, which evaluates not only whether models perform well in development cohorts but also whether they can be successfully integrated into routine clinical and community workflows [24]. Frameworks such as RE-AIM (Reach, Effectiveness, Adoption, Implementation, Maintenance) provide a structured rubric for assessing model-deployment maturity across these dimensions and, together with Technology Readiness Level (TRL)-style staging, are increasingly used to characterize the translational state of prediction-model research [18,19,24].

Despite these methodological advances, existing systematic reviews of ML-based frailty models have mainly summarized algorithms and discrimination metrics, with less attention to practical considerations for public health use, including task definition, predictor burden, external validation, calibration, explainability, and implementation strategy [11,18,19]. These factors are important because models developed for current-status frailty classification may not have the same meaning as models predicting future frailty risk, and high AUROC values may not translate into scalable or actionable screening tools.

This systematic review therefore synthesizes ML-based frailty prediction and classification studies in community-dwelling older adults, with particular attention to methodological quality and public health applicability. We summarize frailty definitions, prediction targets, data sources, predictor domains, algorithms, validation approaches, model performance, explainability methods, and reported deployment strategies across 14 studies published between 2020 and 2025 [25,26,27,28,29,30,31,32,33,34,35,36,37,38]. The goal is not only to compare model performance, but also to clarify the extent to which current models are interpretable, externally validated, and practically usable for community-level frailty screening [14,16,17,18,19].

## 2. Materials and Methods

### 2.1. Study Design

This systematic review was conducted in accordance with the PRISMA 2020 guidelines [39]. The review protocol was prospectively registered in PROSPERO (registration number: CRD420251081555). Principles for the synthesis and reporting of prediction model performance followed established methodological guidance for prognostic and diagnostic model research [14,15,16,18,19].

### 2.2. Search Strategy and Information Sources

The objective of this review was to identify studies that applied machine learning (ML) to predict or classify frailty among community-dwelling older adults and to evaluate the potential contribution of these models to public health practice and intervention strategies [11,18,19]. An initial comprehensive literature search was conducted on 4 July 2025, using PubMed, Embase, Web of Science, and Scopus, covering publications from 1 January 2015 to 30 June 2025. In response to peer-review feedback received during revision, a supplementary search of IEEE Xplore (Journals only) and ACM Digital Library (Research Articles) was conducted in May 2026 using the same search concepts and covering the same period as the primary search (January 2015–May 2026). For IEEE Xplore, the earliest available year option was 2018 because no records matching the search concepts had been indexed in IEEE Xplore before that year; for ACM Digital Library, the full 2015–2025 range was selectable. The search strategy was structured around three conceptual domains—frailty, machine learning, and community-dwelling older adults. Within each domain, terms were combined using the OR operator, and the three domains were combined using AND.

Key search terms included:

- Frailty: “Frailty,” “Pre-frailty,” “Cognitive frailty,” “Physical frailty”.
- Machine learning: “Machine learning,” “Artificial intelligence,” “Deep learning,” “Predictive model,” “Prediction model,” “Classification model”.
- Population: “Community-dwelling older adults,” “Community-living older adults,” “Community-dwelling elderly,” “Older adults living in the community”.

Only peer-reviewed journal articles published in English were included, while reviews, protocols, conference proceedings, books, and trade journals were excluded. Eligible studies were required to involve human participants and to be fully published articles, and studies based on hospital-derived data were excluded. The detailed search strategies for each database are provided in Supplementary Table S1.

### 2.3. Eligibility Criteria and Study Selection

Studies were included if they met all of the following criteria:

1. Participants were community-dwelling adults aged 60 years or older.
2. The study developed or applied ML-based models for frailty prediction or classification.
3. The article was an original, full-length research paper.
4. The sample size was 1000 participants or more.
5. The study was published in a peer-reviewed journal indexed in the Web of Science Core Collection (SCIE, SSCI, or ESCI).
6. Frailty was measured using a validated frailty assessment instrument, such as Fried phenotype-based criteria, Frailty Index approaches, the FRAIL scale, SOF Index, Tilburg Frailty Indicator, Clinical Frailty Scale, or related validated frailty frameworks.

Studies were excluded if they were hospital-based, included participants younger than 60 years, were not original research articles, were published in languages other than English, or were not indexed in the Web of Science Core Collection. The minimum sample size threshold of 1000 participants was prespecified to ensure a sufficient number of outcome events for stable prediction model development and evaluation in community-dwelling populations [40,41]. Frailty is generally a low-prevalence outcome, and smaller samples are prone to unstable discrimination and calibration estimates, as well as increased overfitting during internal validation [21,40,41,42,43]. In rare-event settings, conventional logistic regression and related prediction models may yield biased probability estimates and unstable coefficients when event counts are limited, further underscoring the need for adequate sample sizes and robust validation strategies [44]. Prior methodological studies and reporting guidelines emphasize the importance of adequate event counts for reliable ML-based prediction modeling [14,18,19,40,41]. Accordingly, the ≥1000 sample size criterion was applied as a pragmatic methodological standard to enhance model stability and transportability [18,41].

**Figure 1:**
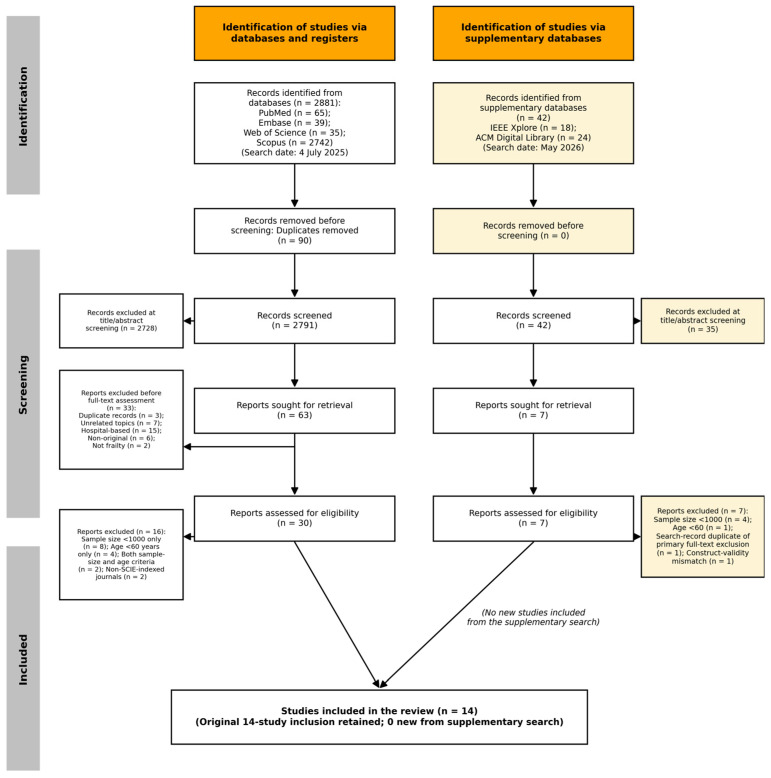
Flow chart of the study selection process. The main PRISMA flow covers the primary searches of PubMed, Embase, Web of Science, and Scopus (executed 4 July 2025). A supplementary search of IEEE Xplore and ACM Digital Library was conducted on 12 May 2026 (42 records identified; 7 assessed at full-text eligibility; 0 new eligible studies). The supplementary-search PRISMA-stage summary is provided in Supplementary Table S6, per-record exclusion reasons for the seven IEEE/ACM full-text candidates are provided in Supplementary Table S7, and the complete 42-record screening log is provided in Supplementary File S1.

All retrieved records were imported into EndNote 2025.3.1 (Clarivate, Philadelphia, PA, USA), where duplicates were removed and automated filters were applied. Of the 2881 records initially identified, 90 duplicates were removed, leaving 2791 records for title and abstract screening. After 2728 records were excluded at the title and abstract screening stage, 63 reports were retained for further assessment. Of these 63 reports, 33 were excluded before full-text eligibility assessment because they were additional duplicate records (n = 3), studies on unrelated topics (n = 7), hospital-based studies (n = 15), non-original articles (reviews, protocols, etc.; n = 6), or not related to frailty (n = 2). The remaining 30 reports underwent full-text assessment. Study selection was primarily conducted by one reviewer (S.K.), and the final inclusion of all eligible studies was independently verified by the second reviewer (J.-H.P.); any uncertainties were resolved through discussion, and no disagreements arose regarding the final set of included studies. Following full-text assessment, 16 reports were excluded because of sample sizes below 1000 only (n = 8), inclusion of participants younger than 60 years only (n = 4), both sample-size and age criteria (n = 2), or publication in journals not indexed in the Web of Science Core Collection (n = 2). Ultimately, 14 studies met all eligibility criteria and were included in the final synthesis (Figure 1) [25,26,27,28,29,30,31,32,33,34,35,36,37,38]. In response to peer-review feedback during revision, a supplementary search of IEEE Xplore and ACM Digital Library was performed in May 2026 using the same search concepts and the same prespecified eligibility criteria. This search identified 42 additional records (IEEE Xplore, n = 18; ACM Digital Library, n = 24). All 42 records were screened at the title/abstract stage; no records were removed before screening. Thirty-five records were excluded at title/abstract screening, including one record that was both a systematic review and a search-record duplicate of a previously screened primary-search record. Seven records were retained for full-text examination. All 7 were excluded at the eligibility stage: 4 for sample sizes below 1000, 1 for inclusion of participants younger than 60 years, 1 search-record duplicate, and 1 on grounds of construct validity, as frailty was operationalized as a surrogate outcome rather than measured via a validated frailty assessment instrument (see Discussion Section 4.5 and Supplementary Table S7 for per-record details) [45]. The supplementary search therefore yielded no additional eligible studies, and the original 14-study inclusion was retained (Figure 1, revised). The PRISMA-stage summary of the supplementary IEEE Xplore and ACM Digital Library search is provided in Supplementary Table S6, the per-record exclusion reasons for the seven IEEE/ACM full-text candidates are provided in Supplementary Table S7, and the complete 42-record screening log is provided in Supplementary File S1 and in the openly accessible GitHub repository (https://github.com/delic1758/ml-frailty-systematic-review-materials, accessed on 27 May 2026).

### 2.4. Data Extraction and Synthesis

Two reviewers (S.K. and J.-H.P.) independently extracted data using a structured template, and discrepancies were resolved through discussion and consensus [14,18,19]. Of 504 extracted data points (14 studies × 36 variables), discrepancies were identified in 8 instances (1.6%), primarily involving predictor counts and reporting of prevalence estimates; all were resolved by consensus. Extracted items included author, publication year, country, study design, sample size and participant characteristics, frailty definition and labeling strategy, data sources, predictors and predictor groupings, ML algorithms, methods for handling class imbalance, missing data strategies, validation approaches (internal and/or external), performance metrics (e.g., AUROC, AUPRC), calibration assessment, use and type of explainable AI (XAI) methods, implementation strategies (web-based tools, scoring systems, EMR [electronic medical record] integration), and reported public health implications [14,18,19,23]. To evaluate predictor-level evidence, we extracted feature-importance information, SHapley Additive exPlanations (SHAP)-based rankings, and variable contribution analyses when available [22,23]. Predictors were subsequently categorized into (1) shared core predictors, (2) trends by predictor domain, and (3) differences across frailty definitions [7,8,9,25,26,27,28,29,30,31,32,33,34,35,36,37,38,46]. We distinguished formal XAI methods from other predictor-level reporting. SHAP and SAGE (Shapley Additive Global Importance) were classified as formal XAI methods. Other reported approaches, including SESv-based biomarker signature extraction, Gini importance, Boruta, RF/XGBoost relative importance, mean decrease accuracy, and RFE-based feature selection, were extracted as predictor-level information or feature-selection/importance reporting rather than treated as formal XAI unless the original article explicitly framed them as explanation methods [20,22,23]. Studies that did not report any form of variable importance, contribution analysis, or selected-feature information were classified as providing no predictor-level evidence [20,22].

To ensure consistent extraction and reporting, three operational terms were defined as follows. (i) Formal XAI methods denote model- or algorithm-agnostic methods producing local and/or global explanations that the original authors framed as explanation methods (e.g., SHAP, SAGE). (ii) Predictor-level information denotes any reporting of feature importance, contribution, selection, or biomarker signature not framed by the original authors as a formal explanation method (e.g., Gini importance, mean decrease accuracy, LASSO, Boruta, recursive feature elimination [RFE], SESv biomarker signatures). (iii) Model deployment denotes any operationalization of the developed model beyond the research environment, including a web calculator, nomogram, point-based risk score, EMR integration scenario, or prospective screening tool. These definitions were applied consistently during data extraction, XAI classification, and implementation-readiness assessment.

Due to substantial heterogeneity across studies in frailty definitions, ML algorithms, performance metrics, and validation strategies, quantitative meta-analysis was not performed [16]. Instead, a narrative synthesis was conducted across study characteristics, frailty definitions, predictor domains, model performance, validation strategies, explainability, and implementation readiness [16]. The synthesis and interpretation of prediction model performance followed methodological recommendations proposed by Debray et al. (2017) [16]. Risk of bias and applicability were assessed independently by two reviewers (S.K. and J.-H.P.) using the Prediction Model Risk of Bias Assessment Tool (PROBAST) [17]. Disagreements were resolved through discussion and consensus. Of 98 domain-level judgments (14 studies × 7 domains), agreement was reached on 89 (90.8%), with disagreements concentrated in the Predictors and Analysis domains; Cohen’s kappa was 0.82, indicating substantial inter-rater agreement. Reporting quality was evaluated using the Transparent Reporting of a Multivariable Prediction Model for Individual Prognosis or Diagnosis (TRIPOD) checklist [14].

Implementation readiness was additionally assessed post hoc using an implementation-science framework, RE-AIM (Reach, Effectiveness, Adoption, Implementation, Maintenance), in combination with a Technology Readiness Level (TRL)-style maturity rubric [18,19,24]. The systematic literature search was not structured a priori around RE-AIM criteria; rather, the RE-AIM and TRL rubric was applied to the 14 included studies during data extraction to characterize the translational state of the field. Operational criteria for each RE-AIM domain were defined as follows: Reach was credited when a study reported a study population consistent with the prespecified eligibility criteria of community-dwelling adults aged ≥60 years and a development cohort of ≥1000 participants. Effectiveness was credited when a study reported model discrimination (AUROC or equivalent) in development and/or validation samples, and, where reported, calibration metrics. Adoption was credited when a study reported either (a) an explicit deployment artifact beyond the development model—such as a web-based calculator or risk scoring tool, a nomogram, or an EMR-integration concept—or (b) an explicitly proposed implementation use case for the developed model in a routine clinical or community-screening setting (e.g., a documented prospective-screening workflow proposal, a primary-screening framing, or a biomarker-signature approach intended for downstream operational use). To preserve interpretability, Table 1 distinguishes concrete deployment artifacts from proposed implementation use cases in the cell text. Implementation was credited when a study reported workflow integration in routine practice, prospective use in a clinical or community setting, or human-factor testing of the deployment artifact. Maintenance was credited when a study reported a post-deployment recalibration plan, drift-monitoring strategy, or documented post-deployment performance. The TRL-style rubric was applied as follows: internal validation only was assigned TRL 4 (laboratory validation); same-cohort temporal validation, TRL 5; independent external validation combined with a deployment artifact, TRL 6 (demonstration in a relevant environment); demonstration in an operational clinical environment, TRL 7 or higher. Because the systematic search did not explicitly target RE-AIM constructs, a lack of reporting in the included studies should be interpreted as absence of evidence rather than evidence of absence; this constraint is acknowledged in Section 4.5.

## 3. Results

### 3.1. Characteristics of Included Studies, Data Sources, and Predictor Composition

A total of 14 studies met the inclusion criteria [25,26,27,28,29,30,31,32,33,34,35,36,37,38]. By country, China accounted for the majority with nine studies (64.3%), followed by one multicountry European study, and one study each from the United Kingdom, Taiwan, Korea, and Thailand (Table 2) [25,26,27,28,29,30,31,32,33,34,35,36,37,38]. Publication years ranged from 2020 to 2025, with most studies published in 2024–2025 (n = 10, 71.4%), indicating a strong concentration of recent research [25,26,27,28,29,30,31,32,33,34,35,36,37,38]. Based on the development or training cohorts, sample sizes ranged from 1230 to 86,133 participants [25,26,27,28,29,30,31,32,33,34,35,36,37,38]. Based on the analytic sample sizes reported in Table 3, the largest sample-size categories were >5000 participants and 2001–3000 participants, each comprising five studies (35.7%) [25,26,27,28,29,30,31,32,33,34,35,36,37,38].

Most studies focused on community-dwelling older adults aged ≥60 years, although some restricted inclusion to those aged ≥65 or ≥70 years [25,26,27,28,29,30,31,32,33,34,35,36,37,38]. Eleven studies (78.6%) used national-level aging cohorts or large administrative datasets, including CHARLS and CLHLS (China), ELSA (UK), KFACS (Korea), and the NHIRD (Taiwan) [25,26,27,28,29,30,31,32,33,34,35,36,37,38]. The remaining three studies (21.4%) relied on community-based survey data: two used locally collected questionnaires and physical assessments (Qi; Isaradech), and one study (Gomez-Cabrero) analyzed stored biospecimens from multiple European cohorts, incorporating multi-omics data [26,36,38]. Half of the studies used longitudinal or prospective cohort designs (n = 7, 50.0%), followed by cross-sectional studies (n = 5, 35.7%) [25,26,27,28,29,30,31,32,33,34,35,36,37,38]. One retrospective cohort study and one nested case–control study were also included [25,26].

Regarding frailty operationalization and predictor construction, Peng et al. constructed a Frailty Index (FI) using 38 chronic disease deficits derived from ICD-9 codes in Taiwan’s national insurance claims database [25]. Gomez-Cabrero et al. integrated genomic, proteomic, metabolomic, and miRNA data with clinical and functional variables across cohorts from Spain, France, and Italy [26]. Among FI-based studies, Huang et al. constructed FI scores using approximately 40–46 accumulated deficits [32]. In some studies, subsets of FI components—primarily functional items such as ADL/IADL (activities of daily living/instrumental activities of daily living) and mobility—were selected and reduced to around 10 predictors depending on analytic objectives [32,37]. In contrast, Dong et al. defined frailty using a 46-deficit FI but limited predictors to nine variables, including age, log-transformed BMI, MMSE, sex, education, occupation, smoking status, and dental health (Table S3) [37]. While this approach preserves conceptual alignment with the FI framework, overlap between outcome definition and predictors warrants caution in performance interpretation [8,13,37]. The remaining studies constructed prediction models using combinations of sociodemographic characteristics, health behaviors, chronic disease history, ADL/IADL, physical performance (e.g., grip strength, gait speed, TUG), cognitive function, depressive symptoms, nutritional status, anthropometry and body composition, oral health, and environmental or social resources, resulting in predictor sets ranging from 5 to 57 variables (Table S3) [28,29,30,31,33,34,35,36,37,38].

### 3.2. Definitions of Frailty and Outcome Variable Construction

**Figure 2:**
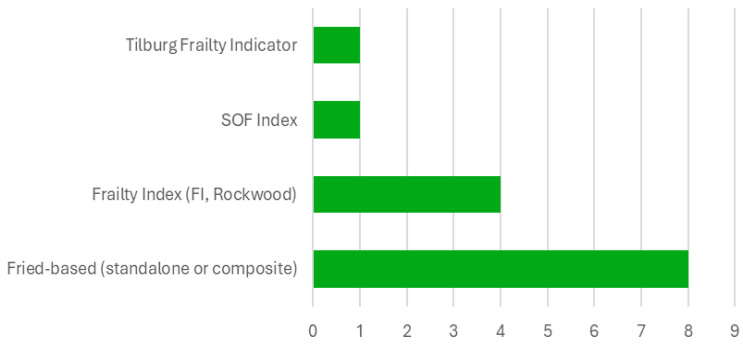
Distribution of frailty definitions among included studies.

Frailty definitions varied substantially across studies (Figure 2) [7,8,9,25,26,27,28,29,30,31,32,33,34,35,36,37,38,46]. Approximately half employed Fried’s physical frailty phenotype or modified versions thereof [7,28,33,34,35,38]. Four studies (about 30%) used a deficit-accumulation Frailty Index (FI) [8,25,27,32,37]. A small number of studies adopted alternative instruments, including the Osteoporotic Fractures (SOF) Index and the Tilburg Frailty Indicator (TFI) (Table 2) [30,36]. Studies applying Fried-based definitions included those by Liu, Du, Hughes, Park, and Isaradech, whereas FI-based studies (Peng, Wu, Huang, and Dong) used continuous indices derived from accumulated functional, disease-related, cognitive, psychological, and sensory deficits [7,8,25,27,28,32,33,34,35,37,38]. Zhang et al. defined frailty using the SOF Index, while Qi et al. employed the TFI [30,36].

Most studies framed frailty prediction as a binary classification problem [25,26,27,28,29,30,31,32,33,34,35,36,37,38]. Overall, 11 studies (78.6%) predicted frail versus non-frail or pre-frail versus non-frail status as a binary outcome [25,26,27,28,29,30,31,32,33,34,35,36,37,38]. Zhang, Du, Qi, and Isaradech included pre-frailty within the outcome definition [30,33,36,38]. Huang and Dong defined frailty using an FI threshold of ≥0.25 [8,32,37]. In contrast, Peng and Gomez-Cabrero applied multi-category or ordinal outcomes (e.g., fit–mild–moderate–severe; robust–pre-frail–frail) [25,26]. Wu et al. modeled frailty trajectories, predicting rapid versus stable FI growth patterns over time [27]. Hughes et al. performed both binary and multiclass classification within the same dataset [34]. Within the cognitive frailty domain, Park et al. defined outcomes by combining Fried phenotype criteria with MMSE scores [7,35]. Liu et al. (2024, 2025) introduced reversible cognitive frailty (RCF) as a distinct outcome, modeling combined physical and cognitive vulnerability as a separate prediction target [29,31].

### 3.3. Key Predictors Identified in Machine Learning-Based Frailty Models

Across studies, several predictor domains were consistently identified as highly influential, including age, burden of chronic conditions or multimorbidity, functional status (ADL/IADL), cognitive function (e.g., MMSE), mental health (particularly depressive symptoms), nutritional and anthropometric measures (BMI, waist or calf circumference), and social factors (Table 3 and Table S3) [4,5,6,25,26,27,28,29,30,31,32,33,34,35,36,37,38]. In FI-based studies, Peng et al. identified hypertension, diabetes, arthritis, heart failure, renal disease, COPD, and depression as major ICD-9-derived deficits contributing to a data-driven modified FI [25]. Huang et al. reported substantial contributions from ADL/IADL deficits, sensory impairments, memory decline, chronic diseases (hypertension and diabetes), and nutrition-related deficits [32]. Dong et al. emphasized a reduced set of predictors, highlighting age, log-transformed BMI, cognitive function (MMSE), sex, education, occupation, smoking status, and dental health [37]. Wu et al. found that age, ADL/IADL, cognitive function, chronic disease burden, and lifestyle factors were key predictors of frailty trajectory classification (rapid versus stable growth) [27].

In SOF-based models (Zhang), age and functional and cognitive measures such as ADL and MMSE emerged as major predictors [30]. In TFI-based models, Qi et al. identified BMI, living arrangements, frequency of visits, and smoking status as the main factors contributing to frailty [36]. Among studies using Fried or modified Fried criteria, predictor importance varied by study design [7,28,33,34,35,38]. In current-status classification models, some studies used predictors overlapping with frailty phenotype components. Hughes et al. included variables closely related to grip strength, gait speed, weight loss, exhaustion, and physical activity, whereas Isaradech et al. selected simpler demographic, anthropometric, disease-related, and exhaustion-related predictors [7,34,38]. In incident frailty risk models using broader predictor sets (Liu 2023; Du), age, BMI or waist circumference, self-rated health, socioeconomic and lifestyle factors, social participation frequency, and oral health were frequently selected as important variables [28,33].

In cognitive frailty studies, Park et al. identified TUG, physical function limitation (PF-M), nutritional status (MNA), K-ADL, and education level as key predictors [35]. Liu et al. (2024) selected 13 predictors for incident reversible cognitive frailty (RCF), including age, education, contact with children, medical insurance, vision impairment, heart disease, medication types, self-rated health, pain locations, loneliness, self-medication, night-time sleep, and running water [29]. Liu et al. (2025) selected five social–ecological predictors: age, medical insurance, self-rated health, SO_2_ exposure, and sunshine duration [31]. Two studies explicitly used formal XAI methods, namely SHAP (Wu; Du) and SAGE (Du), while several others reported feature-importance, biomarker-signature, or feature-selection results: Gini-based importance (Zhang; Huang), LASSO and Boruta (Huang), SESv-based signature extraction (Gomez-Cabrero), RF mean decrease accuracy or RF/XGBoost relative importance (Peng; Liu 2023; Liu 2024; Liu 2025), and RFE-based optimal feature selection (Park) (Table 4) [23,25,26,27,28,29,30,31,32,33,35,36]. The omics-based study by Gomez-Cabrero identified biomarker signatures such as vitamin D3 and troponin T as key predictors, whereas non-omics studies consistently highlighted age, chronic disease burden, functional status, cognitive performance, anthropometric measures, and lifestyle factors [26,27,30,32,33,36].

### 3.4. Applied Machine Learning Algorithms

**Figure 3:**
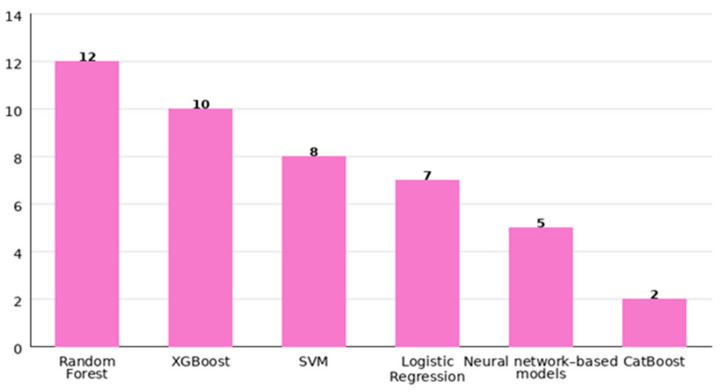
Frequency of machine learning models used across studies.

Most of the included studies compared two or more machine learning algorithms within the same analytical framework (Table 5, Figure 3) [25,26,27,28,29,30,31,32,33,34,35,36,37,38]. Random Forest (RF) and eXtreme Gradient Boosting (XGB) were the most frequently evaluated candidate models, with RF used in 12 studies and XGB in 10 studies [25,26,27,28,29,30,31,32,33,34,35,36,37,38]. Classical machine learning methods—including logistic regression (LR), support vector machines (SVMs), decision trees (DTs), naïve Bayes (NB), and k-nearest neighbors (KNNs)—were commonly included as benchmark models [10,25,26,27,28,29,30,31,32,33,34,35,36,37,38]. In addition, a range of boosting-based approaches such as CatBoost, gradient boosting variants, generalized boosting models (GBM), and CoxBoost were evaluated [25,30,33,34,37]. Several studies also incorporated regression-based models (e.g., modified Poisson regression, generalized linear mixed models) and survival analysis-based approaches (e.g., Cox proportional hazards models, CoxBoost) [15,25,29,31,37].

Neural network-based approaches were applied in a limited number of studies, including those by Wu, Du, Huang, Hughes, and Isaradech, typically in the form of artificial neural networks (ANNs), multilayer perceptrons (MLPs), or shallow learning neural networks (SHLNNs) [27,32,33,34,38]. However, these models were generally treated as part of comparative analyses rather than as primary modeling strategies, and no study implemented modern deep learning architectures [25,26,27,28,29,30,31,32,33,34,35,36,37,38]. Peng et al. used RF for variable selection followed by Cox regression to estimate frailty severity-related risk, while Gomez-Cabrero et al. applied RF and SVM in combination with a meta-analytic framework to identify omics-based frailty biomarkers [25,26]. Du et al. reported that a distilled CatBoost model achieved the highest performance among the compared algorithms, whereas Huang et al., despite evaluating LR, RF, SVM, XGB, SHLNN, and a stacking ensemble, found that LR yielded the highest AUROC [32,33]. Approaches to handling class imbalance varied across studies [25,26,27,28,29,30,31,32,33,34,35,36,37,38]. Four studies explicitly addressed class imbalance using oversampling methods: Wu et al. used SMOTE, Park et al. used SMOTE within bootstrapped training–validation datasets, Hughes et al. used SMOTENC, and Isaradech et al. used SMOTE [27,34,35,38]. Other studies either did not apply imbalance correction or did not report it explicitly [25,26,28,29,30,31,32,33,36,37].

### 3.5. Model Performance: Discrimination (AUROC/AUPRC) and Calibration

Table 5 summarizes the discriminative performance of the prediction models evaluated in the included studies [25,26,27,28,29,30,31,32,33,34,35,36,37,38]. Overall, model discrimination as measured by the area under the receiver operating characteristic curve (AUROC) ranged from moderate to excellent [25,26,27,28,29,30,31,32,33,34,35,36,37,38]. In internal validation, AUROC values ranged from 0.702 for the RF-based frailty trajectory model by Wu et al. to 0.735 for the RF model by Qi et al., 0.756 for the CatBoost model by Du et al., approximately 0.77 for the RF model by Zhang et al., and up to 0.843 for the logistic regression model by Park et al. [27,30,33,35,36]. Studies by Liu et al. (2023, 2024, 2025) reported internal AUROC values ranging from 0.701 to 0.809 [28,29,31]. Notably, Huang et al. and Hughes et al. reported exceptionally high AUROC values for logistic regression models, with internal AUROC of 0.974 and 0.981, respectively [32,34]. Hughes et al. additionally reported an internal AUROC of 0.853 for multiclass frailty classification [34].

For studies that conducted external or temporally distinct validation, both development/internal and external AUROC values were extracted [28,29,30,31,32,34,37,38]. The selected or best-performing models in the Liu et al. studies reported external AUROCs around 0.61–0.62, although model-specific external AUROCs varied and were lower for some algorithms [28,29,31]. Zhang et al. reported temporal external validation across CLHLS waves, with RF and LR achieving external AUROCs around 0.75–0.77 [30]. In the study by Isaradech et al., logistic regression achieved an external AUROC of 0.75, whereas KNN showed substantially lower discrimination (AUROC 0.54) [38]. In contrast, current-status classification or FI-shortening models, such as Huang et al. and Hughes et al., maintained very high external AUROCs above 0.95 [32,34]. Dong et al. reported robust external performance for a Cox regression nomogram, with development AUCs of 0.74, 0.78, and 0.80 at 36, 60, and 96 months and external validation AUCs of 0.68/0.84/0.85 and 0.70/0.72/0.76 across two validation cohorts [37].

Metrics beyond AUROC were reported inconsistently across studies. Selected studies reported accuracy, sensitivity, specificity, precision or recall, F1 or F2 scores, balanced accuracy, Brier scores, or decision-analytic metrics; however, the choice and completeness of these metrics varied substantially across studies [25,26,27,28,29,30,31,32,33,34,35,36,37,38]. None of the included studies reported the area under the precision–recall curve (AUPRC) [25,26,27,28,29,30,31,32,33,34,35,36,37,38]. Calibration-related assessments were reported inconsistently across studies. Several studies reported calibration metrics or plots, including Brier scores, Hosmer–Lemeshow tests, calibration plots, or calibration curves (e.g., Wu; Liu 2023; Liu 2024; Liu 2025; Hughes; Dong; Isaradech), whereas others reported discrimination metrics only [27,28,29,31,34,37,38]. Decision curve analysis or clinical-usefulness assessment was reported in a limited subset of studies, including Wu et al., Liu et al. (2023), Liu et al. (2024), Liu et al. (2025), and Dong et al. [27,28,29,31,37]. A detailed comparison of model performance is presented in Table 5 [25,26,27,28,29,30,31,32,33,34,35,36,37,38].

### 3.6. Validation Strategies and External Validation

Most studies implemented internal validation using data splitting, k-fold cross-validation, or bootstrap resampling (Table 5; Supplementary Table S2) [25,26,27,28,29,30,31,32,33,34,35,36,37,38]. Wu et al., Hughes et al., and Isaradech et al. applied 10-fold cross-validation, while Liu et al. (2023, 2024) used 5-fold cross-validation [27,28,29,34,38]. Park et al. employed 500 bootstrap resamples, and Dong et al. combined 1000 bootstrap resamples with external validation [35,37]. Du et al. applied a hybrid strategy incorporating data splitting, cross-validation, and bootstrapping, whereas Qi et al. conducted a 7:3 train–test split followed by 5-fold cross-validation [33,36]. Despite these approaches, the completeness and transparency of reporting on internal validation procedures and prediction generation varied substantially across studies, as reflected in the TRIPOD checklist assessment (Supplementary Table S5) [14,18,19].

External or temporally distinct validation was reported in eight studies (57.1%): Liu et al. (2023), Liu et al. (2024), Zhang et al., Liu et al. (2025), Huang et al., Hughes et al., Dong et al., and Isaradech et al. [28,29,30,31,32,34,37,38]. Among Chinese cohort studies, external validation was commonly performed using temporally separated survey waves within CHARLS or CLHLS [28,29,31,32]. Zhang et al. developed models using CLHLS wave 6 data and evaluated them using wave 7 data [30]. Hughes et al. conducted temporal external validation by applying a model developed using wave 8 of the English Longitudinal Study of Aging (ELSA) to data from wave 6 [34]. Isaradech et al. evaluated model performance in an independent geographic external cohort from Chiang Mai (n = 464) [38]. These differences should be distinguished from fully independent multi-institutional external validation. In most studies, external AUROC values were lower than those observed in internal validation (e.g., Liu 2023/2024/2025), although performance was maintained or even improved in some cases (e.g., Huang; Dong) [28,29,31,32,37]. Reporting of F1 scores in external validation was limited, precluding firm conclusions regarding generalization performance [28,31,32,34,38]. In contrast, studies by Peng, Gomez-Cabrero, Wu, Du, Park, and Qi did not report external validation, thereby limiting assessment of model generalizability across different populations and data collection contexts [25,26,27,33,35,36].

An additional consideration when interpreting these external validation results is the conceptual distinction between current-status classification and future risk prediction. Studies that classified current frailty status frequently used predictors overlapping with the frailty definition, such as grip strength, gait speed, exhaustion, ADL/IADL limitations, or FI deficit items [32,34,38]. These models should be interpreted as diagnostic or screening classifiers rather than prognostic prediction models, and their high AUROC values—including those reported in external validation—may reflect partial reproduction of the outcome definition itself rather than true forecasting of future risk. In contrast, incident prediction models using temporally preceding predictors generally showed lower external discrimination. The Liu et al. studies reported selected external AUROCs around 0.61–0.62, whereas other prognostic models showed values up to approximately 0.85 depending on the time horizon and validation cohort [28,29,31,37]. This distinction is central to interpreting performance differences across studies and to assessing real-world public health implementation readiness.

### 3.7. Explainability (XAI) and Model Transparency

Two studies explicitly used formal XAI methods. Wu et al. and Du et al. applied SHAP, with Du et al. additionally implementing SAGE [23,27,33]. Several other studies reported predictor-level information rather than formal XAI: Zhang et al. and Huang et al. reported random forest-based Gini feature importance, with Huang et al. further employing LASSO and Boruta for feature selection [30,32]; Qi et al. reported model-derived contributing factors [36]; Gomez-Cabrero et al. proposed core biomarker signatures—such as vitamin D3, troponin T, and multi-omic markers—through SESv-based signature extraction (Table 4) [26]; Peng, Liu 2023, Liu 2024, and Liu 2025 reported RF mean decrease accuracy or RF/XGBoost relative importance [25,28,29,31]; and Park et al. reported RFE-based optimal feature selection [35].

Among studies reporting formal XAI or predictor-level analyses, commonly reported high-importance predictors included age, functional status (ADL/IADL), indicators of chronic disease, and cognitive function (e.g., MMSE) [26,27,30,32,33,36]. Several studies also highlighted physical performance measures (grip strength, gait or walking time), anthropometrics (BMI, waist or calf circumference), and health behaviors or social factors (e.g., smoking status, living arrangements, and visit frequency) [27,32,33,36,38]. However, the set of top predictors varied substantially depending on data modality and prediction targets, particularly in omics- or biomarker-based studies (Table 4; Table 6 or Table S3) [26]. In this review, formal XAI was distinguished from routine feature selection and model-derived importance reporting (Table 4) [20,22,23]. Studies without formal XAI typically reported predictive performance but did not provide systematic individual-level explanation procedures, such as local explanations, counterfactual explanations, or fairness/subgroup interpretability analyses [19,20,22,25,26,27,28,29,30,31,32,33,34,35,36,37,38].

### 3.8. Implementation Strategies and Public Health Applicability

Reported implementation-related elements were summarized across four dimensions: (1) form of deployment, (2) input measurement burden, (3) evidence of operational or prospective validation, and (4) post-deployment monitoring or recalibration plans [18,19]. This descriptive synthesis was used to clarify the maturity and real-world readiness of implementation efforts reported in the included studies [18,19]. With respect to model deployment and implementation, nine of the fourteen studies (64.3%) reported some form of deployment-related output, including nomograms, web-based tools, risk scoring systems, or proposals for EMR integration (Table 4) [25,28,29,30,31,32,33,37,38]. The level of implementation ranged from conceptual suggestions to the presentation of functional web-based prediction tools [25,28,29,30,31,32,33,37,38].

Peng et al. proposed the feasibility of EMR integration for a claims-based frailty index model [25]. Liu et al. (2023) presented a web-based prediction system [28]. Liu et al. (2024) developed a risk scoring tool for reversible cognitive frailty prediction, while Liu et al. (2025) explicitly discussed the use of such scoring tools for public health screening [29,31]. Zhang et al. and Huang et al. presented nomograms incorporating variables such as age, functional and cognitive status, and chronic disease indicators to support risk estimation [30,32]. Huang et al. further combined nomograms with a web-based tool to enhance accessibility of frailty index-based assessment [32]. Dong et al. also implemented a nomogram and evaluated net clinical benefit using decision curve analysis (DCA) [37]. Du et al. used SHAP and SAGE to identify key predictors and reported a risk calculator for estimating pre-frailty risk [23,33]. Isaradech et al. described a web application and discussed EMR integration, outlining workflow scenarios for primary care and community screening [38]. In contrast, five studies (35.7%)—including those by Gomez-Cabrero, Wu, Hughes, Park, and Qi—did not report any deployment strategy [26,27,34,35,36]. Even among studies describing implementation, most approaches remained limited to nomograms, web tools, or scoring systems [28,29,30,31,32,33,37,38]. Within the scope of reporting identified in this review, no study explicitly documented end-to-end production deployment, prospective impact evaluation in real-world settings, or systematic recalibration strategies [18,19,25,26,27,28,29,30,31,32,33,34,35,36,37,38]. Overall, while reporting of implementation strategies is increasing, most efforts remain at a prototype stage [18,19]. For effective public health adoption, further work is needed to develop simplified implementations based on standardized input variables, ensure system integration, conduct external validation and recalibration, evaluate decision utility using methods such as DCA, and establish post-deployment monitoring of outcomes and equity [16,18,19,42].

To formalize this synthesis, we applied the RE-AIM framework (Reach, Effectiveness, Adoption, Implementation, Maintenance) together with a Technology Readiness Level (TRL)-style maturity rubric (Table 6). Reach was documented across all 14 studies through compliance with the prespecified sample size (≥1000) and community-dwelling eligibility criteria. Effectiveness—discrimination performance and, where reported, calibration—was documented across all 14 studies, although the metrics reported varied substantially. Adoption was supported by an explicit deployment artifact in 9 of 14 studies (web calculator, nomogram, risk scoring tool, or EMR-integration concept), whereas 5 studies provided no deployment artifact beyond the model itself. None of the 14 studies provided documented evidence on Implementation (workflow integration, prospective use, or human-factors testing) or Maintenance (recalibration plans, drift monitoring, or post-deployment performance). On the TRL-style rubric, models with internal validation only were placed at TRL 4 (laboratory validation), those with same-cohort temporal validation at TRL 5, and those with independent external validation combined with a deployment artifact at TRL 6 (demonstration in a relevant environment). No study reached TRL 7 or higher (demonstration in an operational clinical environment).

### 3.9. Handling of Missing Data

Across the 14 community-based frailty studies included in this review, strategies for handling missing data were highly heterogeneous [25,26,27,28,29,30,31,32,33,34,35,36,37,38]. Many studies applied complete-case analysis or excluded participants with missing values in key predictors or with a predefined proportion of missing items within the Frailty Index [25,27,32,37]. Several studies also excluded variables when their missingness exceeded a specified threshold. In administrative claims data, imputation was sometimes not performed because deficit-accumulation indices were derived directly from coded diagnoses, and the handling of missing data was insufficiently described in a limited number of studies (Supplementary Table S4). More advanced methods were applied in several studies, most commonly multiple imputation by chained equations (MICE), as reported by Liu et al. (2023) and Hughes et al., the latter combining MICE with variable exclusion (Supplementary Table S4) [28,34]. A small number of studies reported the use of predictive mean matching (PMM) or k-nearest neighbor (KNN)-based imputation, particularly during external validation, while missForest was used in a few instances.

However, explicit justification of the assumed missing data mechanism and alignment between the imputation strategy and the modeling framework were rarely provided [14,18,19]. Overall, sensitivity analyses comparing different missing data approaches were seldom conducted, and reporting practices lacked consistency across studies [14,18,19]. Future research should predefine missing data handling protocols, clearly report missingness patterns, align imputation methods with model structure, and apply identical preprocessing pipelines during external validation to minimize bias that may compromise model transportability [14,18,19].

### 3.10. Risk of Bias and Clinical Applicability

Risk of bias and applicability were assessed for all 14 included studies using the Prediction Model Risk of Bias Assessment Tool (PROBAST) [17]. Domain-specific judgments and overall ratings are summarized in Table 7. For risk of bias, the Participants domain was rated as low risk in 11 studies, high risk in 2 studies, and unclear in 1 study. In the Predictors domain, 6 studies were judged as low risk, 4 as high risk, and 4 as unclear. The Outcome domain was assessed as low risk in 11 studies, high risk in 2 studies, and unclear in 1 study. In the Analysis domain, 6 studies were rated as low risk, 3 as high risk, and 5 as unclear.

Regarding applicability, concern related to the Participants domain was judged as low in 12 studies and high in 2 studies, with no studies rated as unclear. In the Predictors domain, 7 studies showed low concern, 1 study high concern, and 6 studies unclear concern. For the Outcome domain, applicability concern was low in 9 studies, high in none, and unclear in 5 studies. Overall, three studies were classified as having low risk of bias, eight as high risk, and three as unclear. For overall applicability, three studies showed low concern, three showed high concern, and eight were rated as unclear. Only two studies were judged to have both low risk of bias and low applicability concern across all PROBAST domains. Reporting quality was additionally evaluated using the TRIPOD checklist [14], with item-level results for all included studies presented in Supplementary Table S5.

## 4. Discussion

### 4.1. Summary of Key Findings

This systematic review synthesized 14 machine learning (ML)-based studies on frailty prediction or classification among community-dwelling older adults published between 2020 and 2025 [25,26,27,28,29,30,31,32,33,34,35,36,37,38]. Most studies were conducted in East Asian countries, particularly China, and leveraged large-scale national cohorts or administrative databases, including CHARLS, CLHLS, ELSA, KFACS, and NHIRD. Frailty definitions varied widely, encompassing the Fried physical frailty phenotype, the deficit-accumulation Frailty Index (FI), cognitive frailty and reversible cognitive frailty (RCF), as well as the SOF Index and Tilburg Frailty Indicator (TFI). The primary prediction targets were typically binary outcomes, such as frail versus non-frail or pre-frail versus non-frail status.

In terms of modeling strategies, random forest and extreme gradient boosting were most frequently used, alongside logistic regression, support vector machines, CatBoost, gradient boosting methods, Cox-based models, and neural networks. Reported internal validation performance generally ranged from AUROC 0.70 to 0.98. Eight studies reported external or temporally distinct validation; however, the strength of this evidence varied substantially because several validations were conducted within the same parent cohort using different survey waves, whereas only a smaller number used geographically or institutionally distinct populations. External performance often declined in incident prediction models, with AUROCs commonly falling between approximately 0.60 and 0.85. However, current-status classification or FI-shortening models sometimes maintained very high AUROC values, likely reflecting partial overlap between predictors and outcome definitions [27,28,29,30,31,32,34,37,38]. Only a minority of studies explicitly addressed class imbalance, and although several studies reported calibration metrics, Brier scores, decision curve analysis, or clinical-usefulness assessments, these evaluations were inconsistently reported and were not standardized across studies. Formal XAI was explicitly applied in two studies, while several additional studies reported predictor-level importance, biomarker-signature, or feature-selection results, in which age, ADL/IADL deficits, chronic disease burden, physical function, cognitive function, nutritional and body composition indicators, health behaviors, and social or environmental factors consistently emerged as key predictors [26,27,30,32,33,36].

### 4.2. Implications of Heterogeneity in Frailty Definitions and Data Environments

The included studies defined frailty using diverse conceptual frameworks, including FI, Fried phenotype, SOF, TFI, and cognitive frailty or RCF, and employed heterogeneous labeling schemes, such as binary, ternary, ordinal, or trajectory-based classifications (e.g., stable versus rapid progression) [25,26,27,28,29,30,31,32,33,34,35,36,37,38]. This diversity reflects the multidimensional nature of frailty, which encompasses physical function, chronic disease burden, cognitive and mental health, and social or environmental factors. FI-based approaches capture subtle, continuous declines through a deficit-accumulation perspective, whereas Fried-based models emphasize physical performance measures, such as grip strength, gait speed, and unintentional weight loss, yielding a more clinically oriented phenotype. Studies targeting cognitive frailty or RCF explicitly modeled the combined vulnerability of physical and cognitive domains, highlighting an important direction for the conceptual expansion of frailty research.

At the same time, this heterogeneity complicates cross-study comparison and synthesis of model performance. For example, frailty defined as FI ≥ 0.25 and frailty defined by Fried criteria may be similarly associated with adverse outcomes, such as falls, hospitalization, or mortality, yet differ substantially in prevalence and sensitivity–specificity profiles. Even when AUROC values are comparable, differences in baseline prevalence and cutoff structures can lead to markedly different sizes and compositions of the identified frail population. Moreover, FI, Fried, TFI, and SOF capture distinct domains and apply different weighting schemes, implying that studies labeled as “frailty prediction models” may, in practice, be predicting fundamentally different outcomes.

From a data perspective, most studies relied heavily on large Chinese national cohorts, such as CHARLS and CLHLS, with repeated use of a limited number of additional datasets, including ELSA, KFACS, NHIRD, and community health surveys [25,26,27,28,29,30,31,32,33,34,35,36,37,38]. While this concentration has facilitated rapid evidence accumulation for East Asian older populations, it also limits generalizability to Western countries, low- and middle-income settings, and populations with different ethnic, cultural, or health insurance contexts. Furthermore, the majority of datasets consisted of structured survey, examination, or administrative variables. Models incorporating unstructured or high-dimensional data—such as wearable sensor outputs, mobile health data, free-text electronic medical records, or imaging—were notably scarce. This pattern suggests that current ML-based frailty research remains largely within the stage of advanced analysis of traditional tabular data, leaving substantial opportunities for multimodal and longitudinal extensions.

**Figure 4:**
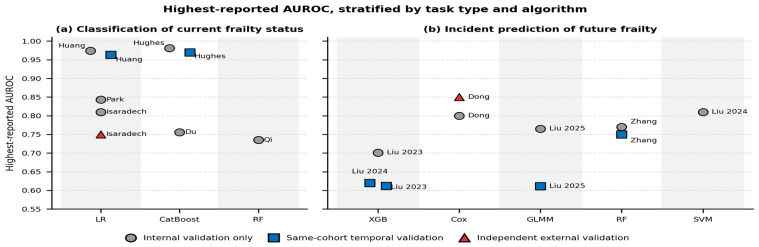
Highest-reported AUROC values for the included machine-learning frailty prediction/classification studies, stratified by task type—(**a**) classification of current frailty status and (**b**) incident prediction of future frailty—and by algorithm class. Points are colored by validation type: internal-only validation (gray circles), same-cohort temporal validation (blue squares), and independent external validation (red triangles). The figure is a descriptive visualization of reported performance and is not a meta-analytic summary; pooled estimates were not computed owing to heterogeneity in frailty definitions, predictor sets, validation strategies, and outcome formulations across studies. For each study, the AUROC plotted is the highest value reported in the study’s primary validation setting (Dong et al. [37]: 96-month AUC in the development cohort and in external validation cohort 1). Two studies (Peng et al. [25]; Gomez-Cabrero et al. [26]) did not report a comparable AUROC and are therefore summarized in Table 5 but not included in this figure. The single trajectory-prediction study, Wu et al. [27], reported an internal AUROC of 0.70 with no external validation, and is not separately plotted; this value is recorded in Table 5. The studies plotted are Huang et al. [32], Hughes et al. [34], Du et al. [33], Park et al. [35], Isaradech et al. [38], Qi et al. [36], Liu et al. (2023) [28], Liu et al. (2024) [29], Liu et al. (2025) [31], Zhang et al. [30], and Dong et al. [37].

Importantly, the included studies addressed two conceptually distinct tasks that should not be conflated. Studies classifying current frailty status frequently used contemporaneous predictors that overlapped partially with the operational frailty definition, raising concerns about circularity and potentially inflated discriminative performance. In contrast, studies predicting incident frailty or trajectory progression used baseline predictors to forecast future risk, providing more directly clinically relevant prognostic information. Throughout the present synthesis (Table 5; Figure 4), classification and prediction studies are therefore reported separately, and AUROC values across these task types are not directly compared.

### 4.3. Interpretation of Modeling Strategies, Performance, and Explainability

Across the studies included in this review, the overall composition of machine learning (ML) algorithms was pragmatic and methodologically conservative. Tree-based ensemble models, such as random forests, extreme gradient boosting (XGB), and CatBoost, were most frequently adopted [25,26,27,28,29,30,31,32,33,34,35,36,37,38]. These models are relatively robust to nonlinear relationships and complex interactions among predictors, and they are well aligned with commonly used explainability tools, including feature importance measures. As such, they represent a reasonable and appropriate choice for frailty prediction. In parallel, traditional statistical models, including logistic regression, generalized linear mixed models (GLMM), and Cox-type models, were also widely applied and, in several studies, demonstrated performance comparable to that of random forests or boosting methods [31,32,34,37]. This finding suggests that a substantial proportion of frailty risk may be explained by predictors with relatively linear effects, such as age, multimorbidity, and functional status, and that increased model complexity does not necessarily guarantee superior performance.

The area under the receiver operating characteristic curve (AUROC) reported for internal validation generally ranged from 0.70 to 0.85, indicating moderate to good discriminative ability [27,28,29,31,33,35,36]. However, several studies reported exceptionally high AUROC values between 0.97 and 0.98 [32,34]. External performance often declined in incident prediction models, with AUROC values commonly falling between 0.60 and 0.85 [28,29,31,37,38]. However, current-status classification or FI-shortening models sometimes maintained very high AUROC values, likely reflecting partial overlap between predictors and outcome definitions. These differences should be interpreted in relation to prediction target, temporal design, and potential overlap between predictors and outcome definitions, and decline was particularly pronounced for simple distance-based algorithms, such as k-nearest neighbors, whose performance deteriorated sharply when applied to independent datasets [38]. These patterns indicate that frailty prediction models can be susceptible to overfitting to cohort-specific distributions and measurement protocols, and that their generalizability may be compromised when applied to different populations or survey waves.

We urge caution when interpreting extremely high AUROC values. In real-world external validation settings, discriminative performance approaching 0.97–0.98 is uncommon. In several studies, frailty outcomes were defined using the Fried phenotype or the Frailty Index, while predictors included overlapping components such as grip strength, gait speed, weight loss, fatigue, or ADL/IADL measures [32,34,38]. Under such conditions, the model may effectively reproduce the frailty definition itself rather than predict future risk, resulting in circularity between predictors and outcomes. This overlap is especially problematic in cross-sectional or same-time-point classification settings, where it can substantially inflate apparent performance. If studies do not distinguish diagnostic classification from prognostic prediction, they may overestimate a model’s real-world applicability. Therefore, direct implementation based solely on high AUROC values from single-cohort or component-overlapping classification models would be premature. Model performance should be interpreted in light of whether components of the frailty definition were included among predictors, the temporal relationship between predictors and outcomes (concurrent classification versus future event prediction), and the intended purpose of the model (diagnostic versus prognostic). At a minimum, external validation in one or more independent cohorts or time periods is essential, and recalibration procedures should be considered when appropriate.

Substantial limitations were also observed in the selection and reporting of performance metrics. Frailty prevalence varies substantially according to definition, population, and setting, ranging from single-digit estimates to substantially higher values in some cohorts, with pre-frailty or early risk states occurring even more frequently [42,43]. This variability has direct implications for class imbalance, metric choice, and the interpretation of AUROC, PPV, and AUPRC. Although this review restricted inclusion to studies with sample sizes exceeding 1000 participants, most studies relied primarily on AUROC and accuracy [25,26,27,28,29,30,31,32,33,34,35,36,37,38]. Metrics that are more informative for rare outcomes, such as the area under the precision–recall curve (AUPRC), sensitivity and specificity, positive and negative predictive values, threshold-dependent trade-offs, and net benefit assessed via decision curve analysis (DCA), were reported inconsistently. This limitation is particularly relevant in frailty research, where outcome prevalence is often low, because discrimination metrics such as AUROC can remain relatively stable despite substantial variation in positive predictive value and precision under rare-event conditions [21]. The use of DCA or clinical-usefulness assessment in a limited subset of studies, including Wu et al., the Liu et al. studies, and Dong et al., provides examples of decision-analytic evaluation, but these analyses were not standardized across studies and were rarely connected to explicit public health threshold-setting or intervention pathways.

From an explainability perspective, studies that applied formal XAI or predictor-level methods such as SHAP, Gini importance, Boruta, SESv, or model-derived feature importance identified age, ADL/IADL, chronic disease burden, cognitive function, physical performance, nutritional or body composition indicators, and health behaviors or social resources as key contributors to frailty prediction [26,27,30,32,33,36]. These findings align closely with established geriatric medicine and public health research on frailty risk factors, thereby reinforcing the face and construct validity of ML-based models. In this sense, ML approaches appear to “rediscover” established knowledge while providing a quantitative assessment of the relative contribution of different domains. Nevertheless, caution is required in interpreting variable importance rankings, particularly in the presence of highly correlated predictors. Importance measures do not imply causal effects, and misinterpretation may lead to inappropriate clinical or policy conclusions. Furthermore, only two studies explicitly used formal XAI methods such as SHAP or SAGE. Although several other studies reported selected features or model-derived predictor rankings, they did not provide systematic explanation of model reasoning or individual-level prediction rationale. For frailty, a multidimensional and cumulative vulnerability, actionable decision-making requires insight into which domains—physical, cognitive, psychological, or social—drive predicted risk and how interventions might be prioritized. Future ML-based frailty research should therefore move beyond performance comparison alone and adopt standardized reporting of predictor-level and domain-level explanations, local explanations at the individual level, and explicit assessments of fairness and subgroup performance. Without these elements, the translation of ML-based frailty prediction models into real-world clinical and public health decision-making will remain limited.

The explainability methods identified in this review fall into four distinct classes, each with different theoretical foundations, scopes of explanation, and implications for clinician trust and clinical adoption. (i) Formal model- or algorithm-agnostic XAI methods (SHAP, SAGE) are grounded in cooperative game-theoretic decomposition of feature contributions; they provide both global and local explanations and are well suited to individual-level decision support, but are computationally intensive and may yield unstable rankings across resampling iterations. (ii) Tree-based feature importance measures (Gini importance, mean decrease accuracy, and relative importance scores from random forest XGBoost, or CatBoost) are computationally efficient and easy to obtain, but provide only global explanations and are known to be biased toward predictors with high cardinality or strong main effects. (iii) Feature-selection approaches (LASSO, Boruta, recursive feature elimination [RFE]) support model parsimony and reproducibility but are not, strictly, explanation methods, because they identify which predictors to retain rather than explain how the model uses them. (iv) Domain-specific biomarker signature extraction (e.g., SESv) is informative within a particular biomedical context but is not directly transferable across cohorts or modalities. Critically, the choice among these classes has direct implications for clinician trust and downstream adoption: post hoc explanations have been criticized for potentially providing only the appearance of interpretability without supporting clinically actionable understanding [20]. In the context of frailty prediction—where the intended action is intervention prioritization across physical, cognitive, psychological, and social domains—formal local explanations combined with stability assessment across resampling are likely to be more useful than feature-importance ranks alone.

### 4.4. Implementation Strategies and Public Health Policy Implications

From an implementation science perspective, most machine learning (ML)-based frailty prediction models identified in this review remain at an early stage of translation, characterized primarily by proof-of-concept deployment rather than evaluation of real-world effectiveness and sustainability [18,19]. While a substantial proportion of studies extended beyond model development and performance reporting to propose concrete implementation strategies, these efforts largely focused on technical feasibility rather than comprehensive assessment of implementation readiness within public health systems [18,19]. Specifically, several studies proposed deployment modalities such as web-based applications, risk scoring tools, nomograms, or integration with electronic medical records (EMRs) [25,28,29,30,31,32,33,37,38]. Peng et al. suggested embedding an insurance-claims-based Frailty Index model into EMR systems to enable automated frailty screening [25]. Huang et al., Du et al., and Isaradech et al. further operationalized their models through web calculators, risk scoring tools, or EMR integration scenarios designed to generate frailty risk scores within routine clinical or health examination workflows [32,33,38]. Liu et al. (2024, 2025), Zhang et al., and Dong et al. translated complex ML outputs into nomograms or point-based scoring systems, thereby facilitating potential use in resource-constrained settings such as primary care clinics, community health centers, and home-visit health services [29,30,31,37]. These approaches suggest a gradual shift from purely methodological contributions toward tools intended for real-world use [18,19].

When examined through an implementation science lens, however, several key components required for large-scale adoption were largely absent across studies [18,19]. Specifically, most studies lacked: (1) prospective impact evaluation assessing usability, acceptability, workflow disruption, and cost-effectiveness in real-world settings; (2) formal workflow integration testing; (3) predefined monitoring and recalibration strategies; and (4) systematic assessment of equity and unintended consequences [18,19,25,26,27,28,29,30,31,32,33,34,35,36,37,38]. Moreover, many proposed tools relied on manual data entry and static formats, such as nomograms or standalone web calculators, which limits scalability for population-level screening and real-time integration into public health workflows [18,19]. Even among studies explicitly addressing implementation, most remained at the level of prototype demonstration rather than pragmatic deployment [18,19,25,26,27,28,29,30,31,32,33,34,35,36,37,38]. In addition, downstream actionability was often insufficiently specified [2,18,19]. Explicit linkages between predicted frailty risk categories and concrete interventions were frequently absent, perpetuating a gap between identifying high-risk individuals and delivering coordinated responses [2,5].

Applying the RE-AIM framework (Reach, Effectiveness, Adoption, Implementation, Maintenance) together with a Technology Readiness Level (TRL)-style maturity rubric to the 14 included studies (Table 6) confirms this assessment in a structured manner. All 14 studies provide documented evidence for Reach (eligible community-dwelling populations of ≥1000 participants) and Effectiveness (discrimination performance), and 9 of 14 provide evidence for Adoption through an explicit deployment artifact (web calculator, nomogram, risk scoring tool, or EMR-integration concept). However, none of the 14 studies provide documented evidence for Implementation (workflow integration, prospective use, or human-factors testing) or Maintenance (recalibration plans, drift monitoring, or post-deployment performance). On the TRL-style rubric, the field is concentrated at TRL 4–6 (laboratory validation to demonstration in a relevant environment), with no study reaching TRL 7 or higher (demonstration in an operational clinical environment). This pattern reinforces the conclusion that, despite methodological maturation, ML-based frailty prediction has not yet been integrated into operational public health workflows.

From a public health and policy perspective, the potential value of ML-based frailty prediction remains substantial [1,5]. Frailty is strongly associated with high-cost and high-burden outcomes, including falls, hospitalization, long-term care utilization, and mortality [1,4,5]. Early identification of high-risk individuals at the community level, coupled with timely interdisciplinary interventions, has the potential to improve population health outcomes while optimizing healthcare and long-term care expenditures [2,5]. Notably, many models reviewed here achieved at least moderate discriminative performance using low-burden inputs derived from surveys, routine health examinations, or administrative data [25,28,30,33,36,37,38]. This characteristic is particularly advantageous for large-scale screening programs [1,6]. Nevertheless, several risks must be carefully managed when integrating frailty prediction models into public health policy [1,19,20]. Systematic under- or overprediction in specific subgroups may exacerbate existing health inequities or misallocate limited resources [19,20]. The use of frailty risk scores to prioritize services also raises concerns about algorithm-mediated discrimination if governance mechanisms are insufficient [19,20]. Furthermore, labeling individuals as high risk without ensuring access to meaningful follow-up interventions may generate psychological distress and stigma without tangible benefit [1,5]. To address these challenges, future studies should explicitly incorporate implementation science frameworks, such as RE-AIM (Reach, Effectiveness, Adoption, Implementation, and Maintenance), to evaluate not only predictive performance but also population reach, real-world effectiveness, adoption, fidelity, and long-term sustainability [24]. Embedding equity assessments, ethical safeguards, and participatory co-design approaches will be essential to support responsible and effective deployment of ML-based frailty prediction tools in real-world public health systems [19,20].

A further consideration for real-world deployment is the computational complexity and deployment-hardware requirement of the underlying algorithms, which varied substantially across the included studies. Logistic regression, decision trees, and shallow ML models impose minimal training and inference cost and can be deployed on low-resource hardware such as single-CPU workstations or point-of-care devices, making them well suited to offline scoring tools, nomograms, and primary-care or community-health settings. Tree-ensemble methods (random forest, XGBoost, CatBoost) incur moderate training cost but offer fast, lightweight inference, supporting web-based calculators and EMR-embedded scoring with minimal latency. Neural-network architectures (artificial neural networks, deep-learning models) impose non-trivial training cost—GPU acceleration is recommended for large input dimensionality or multimodal data—and have a larger memory footprint, although inference cost is typically negligible after pre-training; this becomes important mainly for retraining, recalibration, and federated-learning scenarios in deployed pipelines. Notably, none of the 14 included studies reported computational cost, training time, or deployment-hardware requirements. This represents an additional reporting gap that limits informed selection of algorithms for low-resource and rural community settings, where stable internet, GPU access, and ongoing technical support cannot be assumed.

### 4.5. Strengths and Limitations of This Review

This systematic review has several strengths that distinguish it from prior reviews of ML-based frailty research [11]. First, it focuses specifically on community-dwelling adults aged 60 years and older and deliberately excludes hospital-based studies, thereby prioritizing evidence that is directly relevant to frailty screening and intervention targeting in public health and primary care settings [1,6]. Second, recognizing frailty as a relatively infrequent outcome, we applied a stringent inclusion criterion of a minimum sample size of 1000 participants to reduce the risk of severe model instability and bias arising from insufficient event counts [21,40,41,42,43]. Third, beyond summarizing algorithms, performance metrics, and validation strategies, we systematically extracted and categorized information on explainable AI (XAI) methods and deployment or implementation strategies [18,19]. This approach allowed us to address not only whether models perform well, but also the extent to which they are interpretable and practically usable in real-world contexts [18,19,20,22]. Fourth, in response to peer-review feedback during revision, we performed a supplementary search of IEEE Xplore and ACM Digital Library to extend coverage to engineering and computing venues, and we refined the eligibility criteria to require a validated phenotypic frailty assessment instrument, thereby strengthening construct consistency across the synthesis.

Several limitations should also be acknowledged [11,16]. First, this review intentionally restricted inclusion to English-language articles published in peer-reviewed journals indexed in the Web of Science Core Collection (SCIE, SSCI, or ESCI) to ensure consistency in peer-review standards and reporting practices; consequently, relevant studies published in local journals, non-English languages, conference proceedings, or gray literature were not considered. Second, substantial heterogeneity across included studies in frailty definitions, prediction targets, data sources, algorithms, performance metrics, and validation strategies precluded quantitative meta-analysis, and simple comparisons of summary measures such as AUROC should therefore be interpreted with caution [16]. Third, the review relied on reported performance, calibration, and XAI results, and reproducibility or external validation could not be independently assessed for studies that did not share data or code [14,18,19]. Fourth, due to the rapid evolution of ML and AI research, studies published after the search end date were not included, and the exclusion of preprint servers may have limited capture of the most recent developments [18,19]. Fifth, variation in prediction targets implies differences in event rates and class imbalance across studies, constraining interpretation of performance metrics and inclusion criteria [21,44].

Additional limitations arose from refinements made during the revision stage. Sixth, by requiring a validated phenotypic frailty assessment instrument, we deliberately excluded studies in which frailty was operationalized as a surrogate outcome—for example, 1-year emergency hospitalization or all-cause mortality, as in Bertini et al. (2018) [45]. Although this approach preserves construct consistency and avoids conflating frailty with its adverse outcomes, it may have excluded methodologically relevant work from the broader ML-prediction literature on adverse outcomes in older adults. Seventh, although the supplementary search of IEEE Xplore and ACM Digital Library extended coverage to engineering and computing venues, conference proceedings—frequently the venues at which the most recent algorithmic developments are first reported—were excluded a priori in our PROSPERO-registered protocol, which may have limited capture of cutting-edge ML methods first introduced at major machine-learning conferences. Eighth, the systematic literature search was not structured a priori around the RE-AIM framework; rather, RE-AIM and the TRL-style maturity rubric were applied post hoc, during data extraction, to the 14 included studies. Accordingly, the absence of documented evidence for the Implementation and Maintenance domains in Table 6 reflects a lack of reporting in the included studies and should be interpreted as absence of evidence rather than evidence of absence of these implementation components. Studies may have conducted workflow integration, prospective use, human-factors testing, recalibration, or drift monitoring without explicitly reporting these activities in the publications screened in this review; future implementation-science-oriented systematic reviews should structure search strategies around RE-AIM constructs a priori and consult gray-literature sources (e.g., deployment reports, technical white papers, regulatory submissions) to capture this evidence more completely.

### 4.6. Future Research and Practice Directions

The findings of this review delineate several clear directions for future machine learning (ML)-based frailty prediction research and its application in public health practice [11,18,19]. First, greater standardization of frailty definitions and outcome variables is needed [7,8,9,11,46]. While the coexistence of multiple frameworks reflects the multidimensional nature of frailty, efforts should be made to define shared core indicators whenever possible [4,5,6,46]. At a minimum, studies should explicitly report the frailty definition used, cutoff criteria, prevalence, and the intended clinical or public health target [13,14,17,18,19]. Within a single dataset, parallel development and validation of multiple frailty definitions could further contribute to quantifying conceptual differences between frailty constructs from an ML perspective [9,11,16].

Second, more rigorous methodological standards should be applied across model development, validation, and reporting [14,15,17,18,19]. Future studies should ensure an adequate number of outcome events, particularly in rare-event settings; adhere to prespecified or preregistered analysis plans; and implement appropriate internal validation strategies, such as nested cross-validation [18,19,40,41,44]. Independent external validation, calibration assessment, and decision-analytic evaluations—including decision curve analysis (DCA)—should be considered essential components of frailty prediction research [14,16,17,18,19]. Given the preventive and potentially modifiable nature of frailty, evaluations based solely on discrimination metrics (e.g., AUROC) are insufficient [16,21]. Instead, greater emphasis should be placed on net benefit-based assessments that clarify which populations benefit at which decision thresholds and to what extent [16,37].

Third, explainable artificial intelligence (XAI) and deliberate model simplification strategies should be more actively incorporated [19,20,22,23]. XAI techniques can be used to identify key predictors or predictor domains [20,22,23]. Subsequently, these insights can inform the derivation of low-burden, high-interpretability models, including parsimonious scoring systems and nomograms [29,30,32,33,37]. This two-stage strategy is particularly advantageous for public health implementation [1,6,18,19]. Importantly, the variables identified through this process should be interpreted not merely as contributors to predictive accuracy, but as actionable targets for intervention design [2,5].

Fourth, implementation and impact evaluation must accompany model development [18,19]. Beyond prototyping web-based calculators or electronic medical record (EMR) integrations, frailty prediction tools should be embedded within real-world settings such as primary care and community programs [1,6,18,19]. Prospective studies are needed to evaluate their effects on screening rates, linkage to interventions, hospitalizations, transitions to long-term care, and quality of life [2,5,18,19]. Incorporating implementation science frameworks—such as RE-AIM—would enable assessment of usability, acceptability, ethical considerations, and equity, thereby shifting the focus from isolated model performance to system-level impact [24].

Fifth, diversification of data sources and computational environments represents a critical future challenge [10,12]. To date, most studies have relied on static prediction models using survey, examination, or administrative data [25,26,27,28,29,30,31,32,33,34,35,36,37,38]. Future research should integrate unstructured and continuously generated data with traditional variables [10,12]. Multimodal and continuous monitoring approaches are better suited to capturing the dynamic nature of frailty, enabling detection of trajectories and inflection points rather than single time-point snapshots [10,12]. Such approaches may be particularly valuable for identifying optimal timing for early intervention [2,5]. Finally, the safe integration of ML-based frailty prediction models into public health and clinical decision-making requires not only technical advances but also social consensus and governance [1,19,20]. Ethical and legal frameworks must explicitly address data privacy and security, algorithmic bias and equity, risks of stigmatization or discrimination, and individuals’ rights to autonomy and explanation [19,20]. Within such frameworks, the appropriate role and limitations of frailty prediction tools should be clearly defined [12,19,20]. Expanding predictor extraction to incorporate unstructured clinical narratives via healthcare natural language processing and biomedical named-entity recognition may further enhance ML-based frailty prediction in EHR-rich settings [47].

In summary, ML-based frailty prediction models hold substantial potential to enable early detection of declines in intrinsic capacity among community-dwelling older adults and to support more precise allocation of public health resources [1,2,3,11]. However, translating this potential into tangible health benefits requires maturation toward a trustworthy prediction infrastructure grounded in standardized development, validation, interpretability, and implementation frameworks [14,16,17,18,19,24].

## 5. Conclusions

This systematic review synthesized evidence from 14 machine learning-based studies on frailty prediction and classification among community-dwelling older adults and evaluated the current state of scientific evidence and public health implications [25,26,27,28,29,30,31,32,33,34,35,36,37,38]. Most studies developed models using large-scale datasets with more than 1000 participants, drawn from national aging cohorts or administrative claims data. Across studies, age, burden of chronic diseases, deficits in activities of daily living (ADL) and instrumental ADL, physical and cognitive function, nutritional status and body composition, health behaviors, and social and environmental indicators were consistently identified as key predictors. These findings reaffirm, from a data-driven perspective, the established concept of frailty as a multidimensional and cumulative vulnerability rather than a single disease [4,7,8,46], and they provide important clues for designing multidomain interventions at the population level [1,2,5].

In terms of model performance, most algorithms demonstrated at least moderate discriminative ability in internal validation, and several models based on the Frailty Index or Fried phenotype reported very high AUROC values [32,34]. Performance degradation was frequently observed during external validation of incident prediction models [28,29,31,38]. Moreover, critical elements for assessing model reliability and real-world decision-making suitability—such as handling of class imbalance, calibration, and reporting of AUPRC, decision curve analysis (DCA), and equity or fairness metrics—were inconsistently addressed [16,21]. Formal XAI methods were explicitly used in two studies, while several others reported feature-importance, biomarker-signature, or feature-selection results. Implementation efforts often remained at a prototype or early translational stage, including web-based calculators, risk scoring tools, nomograms, or conceptual proposals for electronic medical record (EMR) integration [18,19,25,28,29,30,31,32,33,37,38]. These limitations indicate that, although ML-based frailty prediction research has moved beyond proof-of-concept, a substantial gap remains before these models can be safely and effectively integrated into real-world public health and clinical systems [11,18,19]. Quantitatively, classification of current frailty status (n = 7) reached AUROCs of 0.70–0.98 with CatBoost (0.97–0.98) and logistic regression (0.96–0.97) as top performers (the latter under same-cohort temporal validation), whereas incident prediction (n = 6) yielded internal AUROCs of 0.70–0.81 and external (same-cohort temporal) AUROCs of 0.58–0.85, and the single trajectory-prediction study (Wu et al.) reported an internal AUROC of 0.70 without external validation. Under PROBAST, only 2 of 14 studies (14.3%) had both low overall risk of bias and low applicability concern, and no study reached a Technology Readiness Level above 6.

Future research should therefore prioritize: (1) clear reporting and, where feasible, standardization of frailty definitions and outcome variables [7,8,9,13,14,46]; (2) adequate event numbers that account for outcome rarity, along with rigorous internal and external validation strategies [17,40,41,44]; (3) multidimensional performance evaluation extending beyond AUROC to include AUPRC, calibration, DCA, and equity-related metrics [16,21]; (4) use of formal XAI and transparent predictor-level reporting to elucidate predictor- and domain-level structures and to develop low-burden yet highly interpretable risk scores [20,22,23]; and (5) technical integration with web, mobile, EMR, and community information systems, accompanied by prospective impact evaluations [18,19,24]. With the establishment of standardized frameworks for model development, validation, interpretation, and implementation, ML-based frailty prediction models have the potential to serve as core tools for early detection of declines in intrinsic capacity among community-dwelling older adults and for the fair and efficient allocation of limited public health resources [1,2,3,6]. This review contributes by clarifying the current position of the field and by delineating priority directions for future research and policy development.

Beyond methodological priorities, future work should explicitly target technical deployment readiness. This entails: prospective in situ evaluation of model performance within primary-care and community-health workflows; standardized reporting of computational complexity, training and inference cost, and deployment-hardware requirements; federated-learning architectures that enable multi-cohort training without centralized data sharing, particularly relevant for cross-jurisdictional aging cohorts; standardized integration interfaces with electronic health record systems and community information infrastructures; predefined recalibration and model-drift monitoring strategies for post-deployment performance maintenance; and explicit governance frameworks addressing fairness, transparency, and clinical accountability. Closing this technical-deployment gap is essential for translating the moderate-to-good AUROC values reported in development cohorts into demonstrable public health impact in community settings.

## Figures and Tables

**Table 1:** Implementation-readiness assessment of included studies using the RE-AIM framework and a Technology Readiness Level (TRL)-style maturity rubric.

Author (Year)	Reach	Effectiveness	Adoption	Implementation	Maintenance	TRL
Peng et al. [25]	Yes	Yes	Proposed EMR integration	NR	NR	TRL 4
Gomez-Cabrero et al. [26]	Yes	Yes	Biomarker-signature approach	NR	NR	TRL 4
Wu et al. [27]	Yes	Yes	Discussed primary-screening use	NR	NR	TRL 4
Liu et al. (2023) [28]	Yes	Yes	Web-based prediction system	NR	NR	TRL 5
Liu et al. (2024) [29]	Yes	Yes	Risk scoring tool with 3 groups	NR	NR	TRL 5
Zhang et al. [30]	Yes	Yes	Actionable nomogram	NR	NR	TRL 5
Liu et al. (2025) [31]	Yes	Yes	Risk scoring with 3 groups	NR	NR	TRL 5
Huang et al. [32]	Yes	Yes	Convenient FI tool	NR	NR	TRL 5
Du et al. [33]	Yes	Yes	Web calculator (proposed)	NR	NR	TRL 4–5
Hughes et al. [34]	Yes	Yes	National-cohort screening framing	NR	NR	TRL 5
Park et al. [35]	Yes	Yes	Community cognitive-frailty screening	NR	NR	TRL 4
Qi et al. [36]	Yes	Yes	Rapid community screening tool	NR	NR	TRL 4
Dong et al. [37]	Yes	Yes	Cox nomogram for individualized risk	NR	NR	TRL 6
Isaradech et al. [38]	Yes	Yes	Web-app and EMR integration design	NR	NR	TRL 6

NR = Not reported in the original publication. RE-AIM domains were credited on the basis of explicit reporting in the included studies; operational criteria for Reach, Effectiveness, Adoption, Implementation, and Maintenance are defined in Section 2.4. Because the systematic search was not structured a priori around RE-AIM constructs, NR should be interpreted as absence of evidence rather than evidence of absence (see Section 4.5). TRL = technology readiness level. TRL 4, laboratory validation (internal validation only); TRL 5, same-cohort temporal validation; TRL 6, independent external validation combined with a deployment artifact (demonstration in a relevant environment); TRL 7 or higher, demonstration in an operational clinical environment.

**Table 2:** General characteristics of the included studies.

Characteristics	Studies (n = 14), n (%)
**Country/region of study**	
China	9 (64.3)
Europe (Spain France Italy)	1 (7.1)
Korea	1 (7.1)
Taiwan	1 (7.1)
Thailand	1 (7.1)
UK	1 (7.1)
**Year of publication**	
2025	6 (42.9)
2024	4 (28.6)
2023	1 (7.1)
2022	1 (7.1)
2021	1 (7.1)
2020	1 (7.1)
**Size of data set**	
1000–2000	2 (14.3)
2001–3000	5 (35.7)
3001–4000	1 (7.1)
4001–5000	1 (7.1)
>5000	5 (35.7)
**Data source**	
National cohorts or administrative datasets	11 (78.6)
Regional/community surveys or multi-cohort datasets	3 (21.4)
**Study design**	
Longitudinal/prospective	7 (50.0)
Cross-sectional	5 (35.7)
Nested case–control	1 (7.1)
Retrospective	1 (7.1)
**Frailty Definition**	
Fried-based (standalone or composite)	8 (57.1)
FI	4 (28.6)
SOF Index	1 (7.1)
TFI	1 (7.1)
**Prediction target**	
Physical frailty only	11 (78.6)
Cognitive frailty or reversible cognitive frailty	3 (21.4)
**Primary prediction type**	
Binary (frail vs. non-frail)	11 (78.6)
Multiclass or ordinal classification (including frailty trajectory analysis)	3 (21.4)

Note: National cohorts or administrative datasets include major longitudinal studies and administrative claims data (e.g., CHARLS, CLHLS, ELSA, KFACS, NHIRD). Regional/community surveys or multi-cohort datasets refer to locally collected questionnaires/physical assessments and multi-cohort biospecimen or omics datasets. Prediction type categories were assigned according to the main analytic target; one study additionally reported multiclass classification [34]. FI = Frailty Index; SOF = Study of Osteoporotic Fractures Index; TFI = Tilburg Frailty Indicator.

**Table 3:** Baseline characteristics of included studies (N = 14).

Author	Country	Data Source	Data Type	Sample Size (N)	Population Description	Study Design
**Peng et al. [25]**	Taiwan	Taiwan National Health Insurance Research Database (NHIRD)	Administrative claims data	86,133 (Fit, Mild, Moderate, Severe frailty)	Community-dwelling adults aged 65–100 years	Retrospective cohort study
**Gomez-Cabrero et al. [26]**	Europe (Spain, France, Italy)	TSHA, AMI, InCHIANTI, 3C-Bordeaux	Biospecimen-based multi-cohort	1522 (Robust 552; Pre-frail 630; Frail 340)	Community-dwelling adults aged ≥65 years	Nested case–control study
**Wu et al. [27]**	China	CLHLS-HF (2002–2018)	Prospective cohort data	4083 (Stable 3370; Rapid 713)	Community-dwelling adults aged ≥65 years	Longitudinal observational study
**Liu et al. (2023) [28]**	China	CHARLS (2011–2015)	Cohort data	Development/internal validation n = 2802 (derivation 2241; internal validation 561); temporal external validation n = 1721	Adults aged ≥60 years	Longitudinal cohort study
**Liu et al. (2024) [29]**	China	CHARLS (national cohort)	Cohort data	2861 total (training 1230; temporal external validation 1631)	Community-dwelling adults aged ≥60 years	Longitudinal cohort study
**Zhang et al. [30]**	China	CLHLS (2011–2014)	Cohort data	6997 (Training 4385; External val. 2612)	Community-dwelling adults aged ≥65 years	Longitudinal cohort study
**Liu et al. (2025) [31]**	China	CHARLS 2011–2013 (training); CHARLS 2013–2015 (external validation)	Cohort data	2861 (Training 1230; external validation 1631)	Community-dwelling adults aged ≥60 years	Prospective cohort study
**Huang et al. [32]**	China	CLHLS (2008, 2011, 2014)	Cohort data + CGA	14,925 (Training 10,672; Internal validation 2667; External validation 1586 [2011: 372; 2014: 1214])	Community-dwelling adults aged ≥65 years	Longitudinal cohort study
**Du et al. [33]**	China	CHARLS (2011–2012)	Cohort data	3141 (Training 2508; Validation 633)	Community-dwelling adults aged ≥60 years	Cross-sectional model development and validation study
**Hughes et al. [34]**	UK	English Longitudinal Study of Aging (ELSA)	Cohort data	5060 (Wave 8 multiclass; Binary 2997; External val. Wave 6: 2002–2218)	Community-dwelling adults aged ≥60 years	Cross-sectional with external validation
**Park et al. [35]**	South Korea	Korean Frailty and Aging Cohort Study (KFACS)	Multi-center cohort	2404 (Cognitive Frailty 443; non-CF 1961)	Community-dwelling adults aged ≥70 years	Cross-sectional study
**Qi et al. [36]**	China	Regional household survey	Survey data	1263	Community-dwelling adults aged ≥60 years (Eastern China)	Cross-sectional study
**Dong et al. [37]**	China	CLHLS (multiple waves)	Cohort data	14,540 (Dev 4878; External val. 1: 3840; External val. 2: 5822)	Community-dwelling adults aged ≥65 years	Longitudinal cohort study
**Isaradech et al. [38]**	Thailand	Community health & anthropometric dataset	Survey + anthropometric data	2692 (Development/internal validation 2228; external validation 464)	Community-dwelling adults aged ≥60 years (Northern Thailand)	Cross-sectional study

Note: N, number of participants; NHIRD, National Health Insurance Research Database (Taiwan); TSHA, Toledo Study for Healthy Aging; AMI, Aging Multidisciplinary Investigation; InCHIANTI, Invecchiare in Chianti; 3C-Bordeaux, Three-City Study (Bordeaux cohort); CLHLS, Chinese Longitudinal Healthy Longevity Survey; CLHLS-HF, Chinese Longitudinal Healthy Longevity Survey—Harmonized Format; CHARLS, China Health and Retirement Longitudinal Study; ELSA, English Longitudinal Study of Ageing; KFACS, Korean Frailty and Aging Cohort Study; CGA, Comprehensive Geriatric Assessment; CF, Cognitive Frailty.

**Table 4:** Summary of XAI application and key predictor analysis in included studies.

Author (Year)	Formal XAI Reported	Reported Method	Predictor-Level Information Reported	Main Predictors or Features as Reported	Predictor Analysis Reported?	Model Deployment/Implementation
**Peng et al. [25]**	No	RF mean decrease accuracy	Yes	38 selected ICD-9 deficits (hypertension, diabetes, arthritis, heart failure, renal disease, COPD, depression)	Yes	EMR-based integration proposed
**Gomez-Cabrero et al. [26]**	No	SESv-based biomarker signature extraction	Yes	Vit D3, Troponin T, omics signatures	Yes	None
**Wu et al. [27]**	Yes	SHAP	Yes	Age, ADL/IADL, chronic diseases, MMSE, body weight, smoking	Yes	None
**Liu et al. (2023) [28]**	No	RF/XGBoost relative importance	Yes	Age, waist circumference, cognitive function	Yes	Web application
**Liu et al. (2024) [29]**	No	RF/XGBoost relative importance	Yes	Age, education, contact with children, medical insurance, vision impairment, heart disease, medication types, self-rated health, pain locations, loneliness, self-medication, night-time sleep, running water	Yes	Risk Scoring Tool
**Zhang et al. [30]**	No	RF Gini importance	Yes	Age, ADL, MMSE, household income, sleep duration, education, housework, meat/fish/egg intake	Yes	Nomogram
**Liu et al. (2025) [31]**	No	RF/XGBoost relative importance	Yes	Age, medical insurance, self-rated health, SO_2_, sunshine duration	Yes	risk scoring tool, public health screening
**Huang et al. [32]**	No	LASSO, Boruta, RF classifier score	Yes	ADL/IADL deficits, chronic disease deficits, cognitive measures	Yes	Web application, Nomogram
**Du et al. [33]**	Yes	SHAP, SAGE	Yes	Living city, BMI, peak expiratory flow (PEF)	Yes	Risk Calculator
**Hughes et al. [34]**	No	No predictor-level explanation emphasized	No or limited	None	No	None
**Park et al. [35]**	No	RFE-based optimal feature selection	Yes	TUG test time, education, PF-M, MNA, ABC, K-ADL	Yes	None
**Qi et al. [36]**	No	Model-derived contributing factors	Yes	BMI, living arrangements, frequency of visits, smoking status	Yes	None
**Dong et al. [37]**	No	Cox nomogram predictors	Yes	Age, log-transformed BMI, MMSE, sex, education, occupation, smoking status, dental health	Yes	Nomogram; DCA reported
**Isaradech et al. [38]**	No	Backward elimination + domain expertise	Yes	Age, gender, household living arrangement, hypertension, dyslipidemia, BMI, waist/calf circumference, exhaustion	Yes	Web application, EMR integration

**Table 5:** Summary of machine learning model characteristics and performance in included studies.

Author (Year)	Task Type	ML Algorithms	Class Imbalance Handling	Validation Type	Validation Strategy	Performance (AUROC)	Other Metrics	Calibration or Overall Performance Metrics Reported
**Peng et al. [25]**	Incident prediction	RF (feature selection), Cox	NR	Internal only	Internal only	NR (HR-based survival model)	HR = 11.4 (Severe vs. Fit)	NR
**Gomez-Cabrero et al. [26]**	Classification	RF, SVM, Meta-analysis	None	Internal only	Internal	NR	OR (Troponin T) = 1.25	NR
**Wu et al. [27]**	Trajectory prediction	RF, LR, NB, DT, SVM, ANN, XGB	SMOTE	Internal only	10-fold CV	Best model (RF): 0.702 (CV); Brier score reported	F1 = 0.816	Brier score; DCA
**Liu et al. (2023) [28]**	Incident prediction	LR, RF, SVM, XGB	None	Same-cohort temporal	External (temporal, CHARLS wave)	Best model (XGB): 0.701 (int), 0.612 (ext)	NR	Hosmer–Lemeshow test, calibration plot, Brier score; DCA
**Liu et al. (2024) [29]**	Incident prediction	Poisson, RF, SVM, XGB	None	Same-cohort temporal	External (temporal)	Internal/training AUC range 0.683–0.809 (best SVM 0.809); external AUC range 0.568–0.620 (best XGB 0.620); MPR-based risk scoring tool	Poisson final model	Hosmer–Lemeshow test, calibration plot, Brier score; DCA
**Zhang et al. [30]**	Incident prediction	RF, SVM, XGBoost, LR	NR	Same-cohort temporal	75:25 split (CLHLS wave 6) + 4-fold CV; temporal external validation (CLHLS wave 7)	Best models RF and LR: AUC~0.77 in pooled final model; external (CLHLS wave 7) AUC~0.75	NR	NR
**Liu et al. (2025) [31]**	Incident prediction	GLMM, SVM, RF, XGB, Binary Mixed Model forest	NR	Same-cohort temporal	External (temporal)	GLMM training AUC 0.765; temporal external validation AUC 0.611; GLMM-based risk scoring tool	F1 not directly reported; sensitivity, specificity, PPV, NPV, and correctly classified rate reported in original article	Hosmer–Lemeshow test, calibration plot, Brier score; DCA
**Huang et al. [32]**	Classification	LR, RF, SVM, XGB, SHLNN, Stacking	None	Same-cohort temporal	10-fold CV + External	Best model (LR): 0.974 (int), 0.963–0.977 (ext)	Acc = 0.932, F1 = 0.880	NR
**Du et al. [33]**	Classification	RF, CatBoost, XGB, ANN	NR	Internal only	80:20 split + 5-fold CV + bootstrap	Distilled CatBoost: AUROC 0.7560 on the 20% holdout test set	Acc = 71.7%, F1 = 0.715	NR
**Hughes et al. [34]**	Classification	CatBoost, GB, LR, RF, KNN, MLP, XGB	SMOTENC	Same-cohort temporal	External (temporal, ELSA wave)	Binary classification: ROC-AUC 0.980–0.981 internally and 0.970–0.971 externally; CatBoost showed best overall performance by F1/Brier-balanced metrics	Multiclass: 0.853 (int), 0.823 (ext)	Brier score
**Park et al. [35]**	Classification	LR + RFE	SMOTE	Internal only	Internal (500 bootstrap)	Best model (LR): 0.843 (bootstrap)	Sens = 75.1%, Spec = 80.9%, Acc = 79.5%	NR
**Qi et al. [36]**	Classification	DT, RF, XGB	NR	Internal only	7:3 split + 5-fold CV	Best model (RF): 0.735 (CV)	F1 = 0.758 (RF), 0.759 (XGB)	NR
**Dong et al. [37]**	Incident prediction	Cox regression nomogram (primary); XGB, GBM, CoxBoost (comparison)	None	Independent external	External (geographic/independent cohorts)	Cox nomogram primary; 36/60/96-month AUCs: development 0.74/0.78/0.80; external validation 1: 0.68/0.84/0.85; external validation 2: 0.70/0.72/0.76	NR	Calibration curve, Brier score; DCA
**Isaradech et al. [38]**	Classification	LR, KNN, RF, MLP, GBC, SVM	SMOTE	Independent external	External (geographic, independent sample)	Best model (LR): 0.81 (int), 0.75 (ext)	Sens/Spec reported	Calibration plot

**Table 6:** Summary of frailty definitions, variable categories, number of predictors, and top predictors in included studies.

Author (Year)	Frailty Definition	Labeling Method (Robust–Pre-frail–Frail)	Variable Categories	Candidate Predictors (n)	Final Predictors Used (n)	Top Predictors
**Peng et al. [25]**	Frailty Index based on cumulative deficit theory	Ordinal (Fit, Mild, Moderate, Severe frailty)	Deficit accumulation (ICD-9 chronic disease deficits)	38	38	Hypertension, diabetes, arthritis, heart failure, renal disease, COPD, depression (38 ICD-9 deficits)
**Gomez-Cabrero et al. [26]**	Fried Frailty Phenotype	Ordinal (Robust/Pre-frail/Frail)	Multi-omics (genomics, proteomics, metabolomics, miRNA) + clinical + ADL/IADL	35,000+ (omics features screened)	2–10	Vitamin D3, lutein/zeaxanthin, miR-125b-5p, cardiac troponin T (non-disabled subgroup: pro-BNP, cardiac troponin T, sRAGE)
**Wu et al. [27]**	Frailty Index (cumulative deficit model)	Binary (stable-growth vs. rapid-growth)	Sociodemographic, lifestyle, economic, ADL/IADL, MMSE, chronic diseases, anthropometrics, childhood starvation	27	27	Physical function, ADL/IADL, marital status, body weight, cognitive function
**Liu et al. (2023) [28]**	Physical frailty phenotype scale	Binary (pre-frail/non-frail)	Sociodemographic, behavior, ADL/IADL, chronic diseases, cognition, sleep, nutrition	46	14	Age, waist circumference, cognitive function (top 3 from 14 LASSO-selected predictors)
**Liu et al. (2024) [29]**	Physical frailty phenotype + subjective cognitive decline (SCD)	Binary (reversible cognitive frailty [RCF]/non-RCF)	Seven social–ecological domains	16	13	Age, education, contact with children, medical insurance, vision impairment, heart disease, medication types, self-rated health, pain locations, loneliness, self-medication, night-time sleep, running water
**Zhang et al. [30]**	Osteoporotic Fractures (SOF) Index	Binary (frail/non-frail)	Sociodemographic, health behaviors, chronic disease history	46	10	Age, ADL, MMSE, household income, sleep duration, education, housework, meat intake, fish intake, egg intake
**Liu et al. (2025) [31]**	Reversible Cognitive Frailty (RCF)	Binary (incident RCF/non-RCF)	Social–ecological domains (final model)	30	5	Age, medical insurance, self-rated health, SO_2_, sunshine duration (5 social–ecological predictors)
**Huang et al. [32]**	Frailty Index (FI ≥ 0.25)	Binary (frail/non-frail)	FI-based 46 deficits (physical, cognitive, psychological, sensory, chronic diseases, function)	46	10	ADL/IADL deficits, sensory impairments, memory decline, chronic diseases (HTN/DM), nutritional deficits
**Du et al. [33]**	Physical frailty phenotype scale	Binary (pre-frail/non-frail)	Sociodemographic, behavior, ADL/IADL, chronic diseases, cognition, nutrition	80	57	Living city, BMI, peak expiratory flow (PEF) (top 3 from distilled CatBoost SHAP)
**Hughes et al. [34]**	Modified Fried Frailty Phenotype	Binary (frail/non-frail) + Multiclass	Modified Fried phenotype + sociodemographic + chronic diseases + ADL/IADL	~20	~20	Grip strength, gait speed, weight loss, exhaustion, physical activity, ADL, depression, BMI
**Park et al. [35]**	Fried Frailty Phenotype + MMSE	Binary (cognitive frailty/non-cognitive frailty)	Sociodemographic, clinical, physical function, psychological, nutrition, falls, motor function	24	6	TUG test time, education level, physical function limitation (PF-M), nutritional status (MNA), balance confidence (ABC), K-ADL
**Qi et al. [36]**	Tilburg Frailty Indicator (TFI)	Binary (frail/non-frail)	Sociodemographic, behavior, diseases, medication, environment, TFI domains	13	13	BMI, living arrangements, frequency of visits, smoking status
**Dong et al. [37]**	Frailty Index (46 indicators)	Binary (frail: FI ≥ 0.25/non-frail)	Sociodemographic, behavior, medical, cognition, oral health, BMI	46 (FI indicators considered)	9	Age, logBMI, MMSE, sex, ethnicity, education, occupation, smoking, dental status
**Isaradech et al. [38]**	Fried Frailty Phenotype	Binary (frail/non-frail)	Sociodemographic, diseases, anthropometric, physical function, fatigue	10	9	Age, gender, household living arrangement, hypertension, dyslipidemia, BMI, waist circumference, calf circumference, exhaustion

**Table 7:** PROBAST risk of bias (ROB) and applicability of ML-based frailty prediction/classification studies in community-dwelling older adults.

Study	ROB: Participants	ROB: Predictors	ROB: Outcome	ROB: Analysis	Overall ROB	App: Participants	App: Predictors	App: Outcome	Overall Applicability
Peng et al. [25]	+	+	+	−	−	+	?	?	?
Gomez-Cabrero et al. [26]	+	+	+	−	−	+	−	+	−
Wu et al. [27]	+	?	−	?	−	+	?	+	?
Liu et al. (2023) [28]	+	+	+	+	+	+	+	+	+
Liu et al. (2024) [29]	+	+	+	+	+	+	?	+	?
Zhang et al. [30]	+	?	+	?	?	+	+	?	?
Liu et al. (2025) [31]	+	+	+	+	+	+	+	+	+
Huang et al. [32]	+	?	−	+	−	+	+	?	?
Du et al. [33]	?	?	+	?	?	+	?	+	?
Hughes et al. [34]	+	−	?	+	−	+	?	?	?
Park et al. [35]	+	−	+	?	−	+	?	?	?
Qi et al. [36]	−	−	+	−	−	−	+	+	−
Dong et al. [37]	+	+	+	?	?	+	+	+	+
Isaradech et al. [38]	−	−	+	+	−	−	+	+	−

The plus symbol (+) indicates low risk of bias (ROB) or low concern regarding applicability (App); the minus symbol (−) indicates high risk of bias or high concern; the question mark (?) indicates unclear risk of bias or applicability.

## Data Availability

No new data were generated in this systematic review. The extracted data, screening records, supplementary search records, PROBAST/TRIPOD assessments, figure source data, and analysis scripts are available in the article, Supplementary Materials, and the public GitHub repository: https://github.com/delic1758/ml-frailty-systematic-review-materials (accessed on 27 May 2026).

## Supplementary Materials

The following supporting information can be downloaded at https://www.mdpi.com/article/10.3390/healthcare14111543/s1, Table S1: Detailed search strategies for each database. Table S2: Validation strategies of included studies. Table S3: Predictor sets and frailty definitions of included studies. Table S4: Missing data handling across included studies. Table S5: TRIPOD checklist assessment for included studies. Table S6: Supplementary IEEE Xplore/ACM Digital Library search PRISMA-stage summary. Table S7: Per-record exclusion reasons for the seven full-text candidates from the supplementary IEEE/ACM search. File S1: Complete 42-record screening log for the supplementary IEEE Xplore and ACM Digital Library search. PRISMA 2020 Checklist.

## Abbreviations

The following abbreviations are used in this manuscript:

Acc	Accuracy
ADL	Activities of Daily Living
AMI	Aging Multidisciplinary Investigation
ANN	Artificial Neural Network
AUPRC	Area Under the Precision–Recall Curve
AUROC	Area Under the Receiver Operating Characteristic Curve
BMI	Body Mass Index
CatBoost	Categorical Boosting
CF	Cognitive Frailty
CHARLS	China Health and Retirement Longitudinal Study
CI	Confidence Interval
CLHLS	Chinese Longitudinal Healthy Longevity Survey
CLHLS-HF	Chinese Longitudinal Healthy Longevity Survey—Harmonized Format
CV	Cross-Validation
DCA	Decision Curve Analysis
DT	Decision Tree
ELSA	English Longitudinal Study of Aging
EMR	Electronic Medical Record
F1	F1 Score (harmonic mean of precision and recall)
FI	Frailty Index
Fried	Fried Frailty Phenotype
GB	Gradient Boosting
GBC	Gradient Boosting Classifier
GBM	Gradient Boosting Machine
GLMM	Generalized Linear Mixed Model
HR	Hazard Ratio
IADL	Instrumental Activities of Daily Living
ICD-9	International Classification of Diseases, 9th Revision
InCHIANTI	Invecchiare in Chianti
KFACS	Korean Frailty and Aging Cohort Study
KNN	K-Nearest Neighbors
LASSO	Least Absolute Shrinkage and Selection Operator
LR	Logistic Regression
MICE	Multiple Imputation by Chained Equations
ML	Machine Learning
MLP	Multilayer Perceptron
MMSE	Mini-Mental State Examination
MNA	Mini Nutritional Assessment
MPR	Modified Poisson Regression
NB	Naïve Bayes
NHIRD	National Health Insurance Research Database (Taiwan)
NN	Neural Network
NPV	Negative Predictive Value
OR	Odds Ratio
PFP	Physical Frailty Phenotype
PMM	Predictive Mean Matching
PPV	Positive Predictive Value
PRISMA	Preferred Reporting Items for Systematic Reviews and Meta-Analyses
PROBAST	Prediction Model Risk of Bias Assessment Tool
RCF	Reversible Cognitive Frailty
RE-AIM	Reach, Effectiveness, Adoption, Implementation, Maintenance
RF	Random Forest
RFE	Recursive Feature Elimination
SAGE	Shapley Additive Global Importance
SCD	Subjective Cognitive Decline
Sens	Sensitivity
SESv	Statistically Significant Signature Variables
SHAP	SHapley Additive exPlanations
SHLNN	Shallow Learning Neural Network
SMOTE	Synthetic Minority Oversampling Technique
SMOTENC	SMOTE for Nominal and Continuous Features
SOF	Study of Osteoporotic Fractures (Index)
Spec	Specificity
SVM	Support Vector Machine
TFI	Tilburg Frailty Indicator
TRIPOD	Transparent Reporting of a Multivariable Prediction Model for Individual Prognosis or Diagnosis
TSHA	Toledo Study for Healthy Aging
TUG	Timed Up and Go
XAI	Explainable Artificial Intelligence
XGB (XGBoost)	eXtreme Gradient Boosting
