# Supplementary material for: Machine Learning-Based Frailty Prediction and Classification in Community-Dwelling Older Adults: A Systematic Review of Validation, Explainability, and Implementation Readiness
Source: Healthcare (Basel). 2026 Jun 1;14(11):1543. doi: 10.3390/healthcare14111543 (PMC13256724; doi:10.3390/healthcare14111543)
Supplement: Supplementary file 1 [file healthcare-14-01543-s001.zip › Supplementary_Materials_with_PRISMA_Checklist_v3.pdf]

Supplementary Materials

Supporting information for: Machine Learning–Based Frailty Prediction and Classification in Community-Dwelling Older Adults: A Systematic Review of Validation, Explainability, and Implementation Readiness

Table S1. Search strategy by database

Database	Exact Boolean search string (with field tags)	Filters/Limits	Number of Records Found
PubMed	("Frailty" OR "Pre-frailty" OR "Cognitive Frailty" OR "Physical Frailty") AND ("Machine learning" OR "Artificial Intelligence" OR "Deep learning" OR "Predictive Model" OR "Prediction Model" OR "Classification Model") AND ("Community-dwelling older adults" OR "Community-living older adults" OR "Community-dwelling elderly" OR "Older adults living in community")	● Date: 01-01-2015 to 30-06-2025<br>● Language: English<br>● Population: Humans	65
Embase	("Frailty" OR "Pre-frailty" OR "Cognitive Frailty" OR "Physical Frailty") AND ("Machine learning" OR "Artificial Intelligence" OR "Deep learning" OR "Predictive Model" OR "Prediction Model" OR "Classification Model") AND ("Community-dwelling older adults" OR "Community-living older adults" OR "Community-dwelling elderly" OR "Older adults living in community")	● Date: 01-01-2015 to 30-06-2025<br>● Document Types: Article<br>● Languages: English<br>● Population(Quick limits): Human	39
Web of Science Core Collection	("Frailty" OR "Pre-frailty" OR "Cognitive Frailty" OR "Physical Frailty") AND ("Machine learning" OR "Artificial Intelligence" OR "Deep learning" OR "Predictive Model" OR "Prediction Model" OR "Classification Model") AND ("Community-dwelling older adults" OR "Community-living older adults" OR "Community-dwelling elderly" OR "Older adults living in community")	● Publication Years(Box check): 2018-2025 (Only year filters were available via box-check options, with no month or day filters applied, and no records were retrieved prior to 2018; furthermore, the search yielded no records published after June 30, 2025.)<br>● Document Types: Article<br>● Languages: English	35
Scopus	("Frailty" OR "Pre-frailty" OR "Cognitive Frailty" OR "Physical Frailty") AND ("Machine learning" OR "Artificial Intelligence" OR "Deep learning" OR "Predictive Model" OR "Prediction Model" OR "Classification Model") AND ("Community-dwelling older adults" OR "Community-	● Search within 'All field'<br>● Publication Years(Box check): 2015-2025<br>● (Only year filters were available via box-check options, with no month or day filters	2,742

	living older adults" OR "Community-dwelling elderly" OR "Older adults living in community")	applied, and no records were retrieved prior to 2019; and after verifying publication dates, studies published after June 30, 2025 were excluded.)<br>● Document Types: Article<br>● Languages: English	
**IEEE Xplore**	("Frailty" OR "Pre-frailty" OR "Cognitive Frailty" OR "Physical Frailty") AND ("Machine learning" OR "Artificial Intelligence" OR "Deep learning" OR "Predictive Model" OR "Prediction Model" OR "Classification Model") AND ("Community-dwelling older adults" OR "Community-living older adults" OR "Community-dwelling elderly" OR "Older adults living in community")	Publication Years (box check): 2018–2025 (the earliest available year option for the search concepts; IEEE Xplore contained no indexed records matching the concepts before 2018). Document Types: Journals only. Languages: English. Supplementary search conducted on 12 May 2026 in response to peer review.	18
**ACM Digital Library**	("Frailty" OR "Pre-frailty" OR "Cognitive Frailty" OR "Physical Frailty") AND ("Machine learning" OR "Artificial Intelligence" OR "Deep learning" OR "Predictive Model" OR "Prediction Model" OR "Classification Model") AND ("Community-dwelling older adults" OR "Community-living older adults" OR "Community-dwelling elderly" OR "Older adults living in community")	Publication Years (box check): 2015–2025. Content Types: Research Articles. Languages: English. Supplementary search conducted on 12 May 2026 in response to peer review.	24

Table S2. Study characteristics, modeling details, and public health implications of included ML-based frailty studies

Study	Country	Study Design	Data Source	Population Description	Sample Size	Frailty Definition	Outcome Type	Machine Learning Methods	Validation Type	Main Performance Metrics	Explainable AI Used	Results Summary	Public Health Implication
**Peng et al.[1]**	Taiwan	Retrospective cohort study	Taiwan National Health Insurance claims data	Older adults aged 65–100 years from Taiwan's National Health Insurance Research Database (NHIRD)	86,133 (Fit/Mild/Moderate/Severe frailty)	Frailty Index (cumulative deficit theory)	Ordinal (Fit / Mild / Moderate / Severe)	Random Forest, Machine learning, Survival analysis, Cox proportional hazards	No external validation reported; RF variable selection and	ML-mFI predicted mortality (HR=11.4), unplanned hospitalizations (HR=6.20), ICU	None reported	ML-mFI outperformed hypothesis-driven mFI for adverse event prediction	ML-mFI shows improved prediction over hypothesis-driven models

									Cox survival analysis	admissions (HR=9.41)		with dose-response patterns	
**Gomez-Cabrero et al.[2]**	Europe	Nested case-control (cross-sectional)	Stored biospecimens (urine, blood, plasma, serum)	Community-dwelling adults ≥65 years from 4 European cohorts (TSHA, AMI, InCHIANTI, 3C-Bordeaux)	1,522 (Robust 552; Pre-frail 630; Frail 340)	Fried Frailty Phenotype	Ordinal (Robust / Pre-frail / Frail)	Random Forests, SVM; meta-analysis; omics biomarker analysis	Internal validation across cohorts (nested case-control)	Protective biomarkers: vitamin D3 OR=0.81, lutein/zeaxanthin OR=0.82, miRNA125b-5p OR=0.73; Risk biomarkers: Troponin T OR=1.25, pro-BNP OR=1.47, sRAGE OR=1.26	SESv	Vitamin D, oxidative stress, and cardiovascular biomarkers associated with frailty	Suggests early detection and personalized intervention using multisystem biomarkers
**Wu et al.[3]**	China	Longitudinal observational study	CLHLS-HF longitudinal cohort	Community-dwelling adults ≥65 years (CLHLS-HF, 2002–2018)	4,083 (Stable 3,370; Rapid 713)	Frailty Index (cumulative deficit model)	Binary frailty trajectory (stable-growth / rapid-growth)	RF, LR, NB, DT, SVM, ANN, XGBoost	10-fold CV; SHAP interpretability	RF AUC=0.702; Precision=0.820; Recall=0.845; F1=0.816	SHAP	Two trajectories identified; key predictors include ADL/IADL, MMSE, marital status, chronic diseases	Enables early identification of high-risk progression for targeted prevention
**Liu et al. (2023)[4]**	China	Longitudinal cohort study	CHARLS 2011–2015 waves	Community-dwelling adults ≥60 years (CHARLS)	Development/internal validation n=2,802 (derivation 2,241; internal validation 561);	Physical frailty phenotype	Binary [(pre)frail / non-frail]	LR, RF, SVM, XGBoost	5-fold CV; internal + temporal external validation	XGBoost AUC=0.701 (internal), 0.612 (external)	Variable importance	Web-based prediction system; Key predictors were identified via variable selection, without	Web tool for early identification and preventive intervention

					temporal external validation n=1,721							an explicit XAI framework.	
**Liu et al. (2024a)[5]**	China	Longitudinal cohort study	CHARLS national longitudinal survey	Community-dwelling adults ≥60 years (CHARLS, 2011–2015)	2,861 total (training 1,230; temporal external validation 1,631)	RCF = Physical frailty phenotype + Subjective cognitive decline	Binary (RCF / non-RCF)	Modified Poisson regression, SVM, RF, XGBoost	5-fold CV; temporal external validation	Internal AUCs: XGB 0.701, RF 0.687, LR 0.676, SVM 0.676; External AUCs: XGB 0.620, RF 0.602, LR 0.617, SVM 0.568	LASSO-based selection; modified Poisson importance	Risk scoring tool with three risk groups; user-friendly for practice	Supports early prediction of RCF and tailored prevention
**Zhang et al.[6]**	China	Longitudinal cohort study	CLHLS national longitudinal survey	Community-dwelling adults ≥65 years (CLHLS, 2011–2014)	6,997 (Training 4,385; External val. 2,612)	SOF Index (3-item)	Binary (frail / non-frail)	RF, SVM, XGB, LR	Internal (75:25 split) + temporal external validation	RF AUC=0.75; LR AUC=0.74; final pooled RF/LR AUC=0.77/0.76; accuracy=87.4%	Feature importance (Gini)	RF and LR selected as final models; predictors included age, ADL, MMSE, income, sleep, education, housework, and diet-related variables	Provides actionable nomogram for early intervention planning
Liu et al. (2025)[7]	China	Prospective cohort study	CHARLS longitudinal cohort	Community-dwelling adults ≥60 years (CHARLS 2011–2013/2013–2015)	2,861 (Training 1,230; external validation 1,631)	Reversible Cognitive Frailty (RCF)	Binary (incident RCF / non-RCF)	GLMM, SVM, RF, XGBoost, Binary Mixed Model forest	5-fold internal validation + temporal external validation	GLMM AUC = 0.765 (train, 95% CI 0.736–0.795), 0.611 (external, 95% CI 0.545–0.725)	RF/XGBoost predictor importance reported; no formal XAI	Five social-ecological predictors selected: age, medical insurance, self-rated health, SO_2, sunshine duration	Supports prevention using multidomain risk assessment

**Huang et al.[8]**	China	Longitudinal cohort study	CLHLS; Comprehensive Geriatric Assessment	Community-dwelling adults ≥65 years (CLHLS 2008/2011/2014)	14,925 (Training 10,672; Internal validation 2,667; External validation 1,586 [2011: 372; 2014: 1,214])	Frailty Index (binary frail / non-frail)	Binary (frail / non-frail)	LR, RF, SVM, XGB, SHLNN, Stacking	Same-cohort temporal validation (CLHLS 2008 training/internal validation; CLHLS 2011 and CLHLS 2014 waves used as same-cohort temporally separated validation sets, not independent	LR AUC≈0.974 internal; external AUC 0.963–0.977; F1>0.88; Acc>0.90	LASSO, Boruta, RF importance	Daily function and physical health measures drive predictions; strong external validation	Convenient FI assessment tool for clinical and public health management

									external cohorts)				
**Du et al.[9]**	China	Cross-sectional model development and validation study	CHARLS	Community-dwelling adults ≥60 years (CHARLS 2011–2012)	3,141 (Training 2,508; Validation 633)	Physical frailty phenotype	Binary (pre-frail / non-frail)	Stacking-CatBoost, CatBoost, RF, XGB, LightGBM, ANN, LR	80:20 hold-out split + fivefold cross-validation + bootstrap	Distilled CatBoost AUROC=0.7560 on the 20% holdout test set; Acc=71.74%; F1=0.715	SHAP, SAGE	Living city, BMI, peak expiratory flow among key factors	Explains pre-frailty risk for actionable screening
**Hughes et al.[10]**	UK	Cross-sectional with external validation	ELSA (internal: Wave 8; external: Wave 6)	Community-dwelling adults aged ≥60 years	Wave 8: binary 2,997; multiclass 5,060; Wave 6 external val.: 2,002–2,218	Modified Fried	Binary and multiclass	CatBoost, Gradient Boosting, Logistic Regression, Random Forest, KNN, MLP, XGBoost	10-fold cross-validation + external validation	Binary: LR AUC=0.981 (internal), 0.971 (external); Multiclass: AUC=0.853 (internal), 0.823 (external)	None	Binary classification: LR/LightGBM achieved the highest ROC-AUC, while CatBoost showed best overall binary performance by recall, balanced accuracy, F1, and Brier score; multiclass classification remained more difficult	Supports large-scale screening using national cohort survey data
**Park et al.[11]**	Korea	Cross-sectional	KFACS	Community-dwelling adults aged 70–84 years	2,404 (Cognitive Frailty 443; non-CF 1,961)	Fried ≥1 + MMSE ≤24	Binary (cognitive frailty vs. non-	Logistic Regression with RFE and bootstrapping	500-bootstrap; SMOTE	AUC=0.843; Sensitivity=75.1 %; Specificity=80.9	None	Stable feature selection with LR+RFE and	Practical for community cognitive-frailty screening

						(cognitive frailty)	cognitive frailty)			%; Accuracy=79.5%		good discrimination	
**Qi et al.[12]**	China	Cross-sectional	Community surveys in Eastern China (Shandong, Jiangsu, Guangdong)	Community-dwelling adults aged ≥60 years	1,263	Tilburg Frailty Indicator	Binary (frail vs. non-frail)	Decision Tree, Random Forest, XGBoost	70:30 split; 5-fold CV	RF AUC=0.735, F1=0.758; XGB AUC=0.720, F1=0.759	Feature importance	RF and XGB showed comparable performance; simple indicators enabled prediction	Usable as a rapid community screening tool
**Dong et al.[13]**	China	Longitudinal cohort	CLHLS	Community-dwelling adults aged ≥65 years	Development cohort: 4,878; External validation cohort 1: 3,840; External validation cohort 2: 5,822	Frailty Index (FI ≥ 0.25)	Binary (frail vs. non-frail)	Cox, XGBoost, GBM, CoxBoost	1,000-bootstrap + external validation	Cox nomogram AUC=0.74–0.80 in development; external AUC=0.68–0.85 across two validation sets	None	Cox nomogram primary model; ML survival models used for comparison; calibration and DCA reported	Provides individualized risk estimates and informs public-health screening design
**Isaradech et al.[14]**	Thailand	Cross-sectional with external validation	Community dataset in Northern Thailand	Community-dwelling adults aged ≥60 years	2,692 (Development /internal validation 2,228; external validation 464)	Fried Phenotype	Binary (frail vs. robust)	Logistic Regression, KNN, Random Forest, MLP, Gradient Boosting, SVM	10-fold CV + external; SMOTE	LR AUC=0.81 (internal), 0.75 (external); KNN AUC=0.85 (internal), 0.54 (external)	None	Logistic regression generalized best; KNN overfit with external performance drop	Designed for web-app and EMR integration in primary care and community screening

Table S3. Characteristics of predictive features in the selected studies.

Study	Feature selection method	Predictor count	Predictive features	Missing Data Handling
**Peng et al.[1]**	Random forest variable importance (data-driven approach)	38	Anemia; Diabetes mellitus; Meniere's disease; Senile dementia; Disorders of fluid, electrolyte and acid-base balance; Cerebral atherosclerosis; Paralysis agitans (Parkinson's disease); Other diseases of lung; Disorders of stomach; Hypertensive heart disease; Disorders of kidney and ureter; Gastroenteritis and colitis; Coronary artery disease; Fractures of intracapsular section of femur; Hypertrophy (benign) of prostate; Paroxysmal supraventricular tachycardia; Streptococcal septicemia; Contact dermatitis and other eczema; Congestive heart failure; Malignant neoplasm of liver; Pruritus; Cerebral thrombosis; Chronic liver diseases; Osteoporosis; Cognitive deficits, late effects of cerebrovascular disease; Intestinal obstruction; Renal colic; Acute, but ill-defined, cerebrovascular disease; Viral hepatitis; Simple chronic bronchitis; Secondary malignant neoplasm of kidney; Bronchial asthma; Streptococcus infections; Chronic airways obstruction; Gouty arthropathy; Diseases of esophagus; Hypertensive chronic kidney disease; Malignant neoplasm of trachea, bronchus, and lung	No imputation; deficits defined by claims algorithm (≥3 outpatient or ≥1 inpatient record)
**Gomez-Cabrero et al.[2]**	Three-step ML pipeline: (1) permutation and meta-analysis for robust screening, (2) SESv (Statistically Equivalent Signature variables) selection with multiple imputations, (3) minimal model search with 2–10	13	25-hydroxyvitamin D3, Lutein/Zeaxanthin, miRNA-125b-5p, Retinol (cohort-specific), Urine peptide 56884, -$CHCH_2CH$- moiety (protective in TSHA cohort)., Cardiac troponin T, pro-BNP, sRAGE, Malondialdehyde (MDA), miRNA-194-5p (InCHIANTI), Citrate (TSHA cohort)	Variables with a high proportion of missing data (over 20%) were excluded in advance, and the remaining missing values were addressed using 1000-fold multiple imputation for each cohort prior to SESv and model development.

	variable combinations using RF and SVM			
**Wu et al.[3]**	Recursive feature elimination (RFE) with 10-fold cross-validation based on random forest	27	IADL, ADL, MMSE, Marital status, Weight, Hypertension, Heart disease, Stroke/cerebrovascular disease, Childhood starvation, Medical cost payer, Education, Economic status, Alcohol consumption, Entertainment activities, Social activity, Arthritis, Residence (urban/rural), Age, Sex, Smoking, Chronic disease status, Self-rated health, Physical activity, Medical service in childhood, Current residence type, plus other demographic and lifestyle indicators included in Supplementary Tables S2 and S5	Variables with missing values imputed using missForest (non-parametric random forest–based imputation for mixed data types)
**Liu et al. (2023)[4]**	LASSO regression with 10-fold cross-validation	14	Waist circumference, Age, Cognitive function, Self-rated health, Material wealth, Medical insurance, Current residence location, Pension insurance, Housing tenure, Afternoon napping, Eating habit, Hearing impairment, Depressive symptoms, Lung disease	Multiple imputation by chained equations for missing candidate predictors
**Liu et al. (2024a)[5]**	LASSO regression	13	Age, Education level, Contact with children, Medical insurance, Vision impairment, Heart diseases, Medication types, Self-rated health, Pain locations, Loneliness, Self-medication, Night-time sleep, Having running water	Multiple imputation by chained equations for candidate predictors
**Zhang et al.[6]**	Clinical relevance + literature review; feature importance ranked by Random Forest	10	Age, Activities of Daily Living (ADL), Mini-Mental State Examination (MMSE), Income, Sleep hours, Education, Housework, Meat, Fish, Egg	Multiple imputation by chained equations (MICE, 5 imputations × 10 cycles)
Liu et al. (2025)[7]	glmmLasso variable selection within GLMM; RF/XGBoost predictor importance also reported	5	Age, Medical insurance, Self-rated health, SO_2 exposure, Sunshine duration	Multiple imputation by chained equations for candidate predictors
**Huang et al.[8]**	LASSO regression + Boruta algorithm + Random forest classifier scoring	10	Able to go shopping by yourself, Able to walk one kilometer, Able to carry 5kg weight, Able to make food by yourself, Able to crouch and stand for three times, Able to wash clothes by yourself, Able to take public transport, Able to go outside to visit neighbors, Able to pick up a book from the floor, Able to stand up from sitting in a chair	Samples with missing values in frailty indicators and demographics were excluded

**Du et al.[9]**	Recursive Feature Elimination (RFE) with LightGBM, followed by stacking-CatBoost distillation	57	Living city, BMI, Peak expiratory flow (PEF), In-house shower or bath facility, Weight, Chest pain during exertion, Age, Falls within last 2 years, Arthritis or rheumatism, Community-based elderly association	Participants with missing data in selected features were excluded from analysis
**Hughes et al.[10]**	Random Forest–based embedded feature importance (retain features contributing to 95% cumulative importance)	Binary: 34; multiclass: 57	Binary and multiclass selected predictors included demographic, health, physical function, chronic disease, and psychosocial variables retained by RF-based feature selection	MICE (Multivariate Imputation by Chained Equations) for missing values; SMOTENC oversampling for class imbalance
**Park et al.[11]**	Recursive Feature Elimination (RFE) with bootstrapping logistic regression	6	TUG test time, Education level, PF-M (physical function limitation), MNA (nutritional status), ABC (balance confidence), K-ADL (activities of daily living)	Participants with incomplete physical frailty and/or cognitive assessment were excluded; missing-data imputation not reported
**Qi et al.[12]**	Univariate logistic regression → multivariate logistic regression → included significant factors ($p<0.05$) into ML models (Decision Tree, Random Forest, XGBoost)	13	Age, BMI, Monthly income, Living arrangement, Visit frequency, Pension insurance, Smoking status, Number of chronic diseases, Type of medication, Sleep duration, Outgoing frequency, Gender, Marital status	Missing or inconsistent records excluded during data cleaning; dataset split into 70% training and 30% testing
**Dong et al.[13]**	The Frailty Index (FI) was constructed using 46 health deficit variables. For the prediction model, nine sociodemographic, behavioral, and medical predictors were selected through stepwise	9	Age, BMI (log-transformed), Cognitive function (MMSE), Sex, Nationality, Education, Occupation, Smoking status, Natural teeth status	Participants with >30% missing FI variables excluded; FI computed using available deficits; internal validation via 1000 bootstrap samples

	multivariate Cox regression.			
**Isaradech et al.[14]**	Multivariable logistic regression with backward elimination (p<0.20) + domain expertise	9	Age, Gender, Household living arrangement, Hypertension, Dyslipidemia, BMI, Waist circumference, Calf circumference, Exhaustion	Internal dataset: complete case analysis (0.18% missing age). External dataset: multiple imputation (PMM, KNNImputer) for age, BMI, waist, calf, handgrip; frailty status missing cases removed

Supplementary Table S4. Handling of Missing Data in Included Studies.

Author (Year)	Missing Data Reported	Primary Handling Strategy	Imputation Method	Exclusion Criteria Related to Missing Data	Sensitivity Analysis
**Peng et al.[1]**	NR	NR	NR	NR	No
**Gomez-Cabrero et al.[2]**	Yes	Variable exclusion + multiple imputation	Multiple imputation (1,000 imputations; method NR)	Variables with >20% missing values excluded	No
**Wu et al.[3]**	Yes	Imputation + FI missing-item handling	missForest	FI: missing items (≤1/3) excluded from numerator/denominator	Yes

**Liu et al. (2023)[4]**	Yes	Multiple imputation	Chained equations algorithm (MICE)	Insufficient frailty measurement excluded; external validation excluded missing data on 14 predictors	Yes
**Liu et al. (2024a)[5]**	Yes	Multiple imputation	Chained equations algorithm (MICE)	Missing age / key outcome-component data and/or insufficient RCF measurement excluded (per flowchart)	Yes
**Zhang et al.[6]**	Yes	Multiple imputation	MICE (R package)	Excluded if lacked component answers to diagnose frailty; died/lost to follow-up	No
Liu et al. (2025)[7]	Yes	Multiple imputation	Chained equations algorithm (MICE)	Missing age / key outcome-component data and/or insufficient RCF measurement excluded (per flowchart)	Yes
**Huang et al.[8]**	Yes	Exclusion / complete-case	None	Excluded samples with missing FI indicators or demographic characteristics; incomplete FI indicator data and/or excessive missing demographic variables excluded	No
**Du et al.[9]**	Yes	Exclusion / complete-case	None	Participants with missing information among selected features excluded	No
**Hughes et al.[10]**	Yes	Variable exclusion + multiple imputation	MICE	Variables with missingness >30% removed	No
**Park et al.[11]**	Yes	Exclusion / complete-case	None	Excluded participants with incomplete physical frailty and/or cognitive function data	No

**Qi et al.[12]**	Yes	Exclusion / complete-case	None	Excluded questionnaires/participants with missing key variables	No
**Dong et al.[13]**	Yes	Exclusion + FI missing-item handling	None	Excluded FI variables missing >30% and baseline-variable missingness; FI computed using available deficits and excluded if >30% deficits missing	No
**Isaradech et al.[14]**	Yes	Complete-case (internal) + multiple imputation (external)	PMM (5-NN; KNNImputer)	External dataset: 4 records with missing outcome removed; missing values imputed (external)	No

- NR indicates not reported.
- Missing data handling strategies were extracted as reported in each original study; no additional assumptions regarding missing data mechanisms (e.g., MAR/MNAR) were made.
- Sensitivity analysis column indicates whether the study explicitly reported sensitivity analyses related to missing data/attrition or robustness checks; otherwise marked "No".

Table S5. TRIPOD reporting quality checklist in the selected studies.

Section/Topic	Item		Checklist Item
**Title and abstract**			
Title	1	D;V	Identify the study as developing and/or validating a multivariable prediction model, the target population, and the outcome to be predicted.
Abstract	2	D;V	Provide a summary of objectives, study design, setting, participants, sample size, predictors, outcome, statistical analysis, results, and conclusions.
**Introduction**			
Background and objectives	3a	D;V	Explain the medical context (including whether diagnostic or prognostic) and rationale for developing or validating the multivariable prediction model, including references to existing models.
	3b	D;V	Specify the objectives, including whether the study describes the development or validation of the model or both.
**Methods**			
Source of data	4a	D;V	Describe the study design or source of data (e.g., randomized trial, cohort, or registry data), separately for the development and validation data sets, if applicable.
	4b	D;V	Specify the key study dates, including start of accrual; end of accrual; and, if applicable, end of follow-up.
Participants	5a	D;V	Specify key elements of the study setting (e.g., primary care, secondary care, general population) including number and location of centres.

	5b	D;V	Describe eligibility criteria for participants.
	5c	D;V	Give details of treatments received, if relevant.
Outcome	6a	D;V	Clearly define the outcome that is predicted by the prediction model, including how and when assessed.
	6b	D;V	Report any actions to blind assessment of the outcome to be predicted.
Predictors	7a	D;V	Clearly define all predictors used in developing or validating the multivariable prediction model, including how and when they were measured.
	7b	D;V	Report any actions to blind assessment of predictors for the outcome and other predictors.
Sample size	8	D;V	Explain how the study size was arrived at.
Missing data	9	D;V	Describe how missing data were handled (e.g., complete-case analysis, single imputation, multiple imputation) with details of any imputation method.
Statistical analysis	10a	D	Describe how predictors were handled in the analyses.
	10b	D	Specify type of model, all model-building procedures (including any predictor selection), and method for internal validation.
	10c	V	For validation, describe how the predictions were calculated.
	10d	D;V	Specify all measures used to assess model performance and, if relevant, to compare multiple models.
	10e	V	Describe any model updating (e.g., recalibration) arising from the validation, if done.
Risk groups	11	D;V	Provide details on how risk groups were created, if done.
Development vs. validation	12	V	For validation, identify any differences from the development data in setting, eligibility criteria, outcome, and predictors.
**Results**			
Participants	13a	D;V	Describe the flow of participants through the study, including the number of participants with and without the outcome and, if applicable, a summary of the follow-up time. A diagram may be helpful.
	13b	D;V	Describe the characteristics of the participants (basic demographics, clinical features, available predictors), including the number of participants with missing data for predictors and outcome.
	13c	V	For validation, show a comparison with the development data of the distribution of important variables (demographics, predictors and outcome).
Model development	14a	D	Specify the number of participants and outcome events in each analysis.
	14b	D	If done, report the unadjusted association between each candidate predictor and outcome.
Model specification	15a	D	Present the full prediction model to allow predictions for individuals (i.e., all regression coefficients, and model intercept or baseline survival at a given time point).
	15b	D	Explain how to use the prediction model.
Model performance	16	D;V	Report performance measures (with CIs) for the prediction model.
Model updating	17	V	If done, report the results from any model updating (i.e., model specification, model performance).

**Discussion**			
Limitations	18	D;V	Discuss any limitations of the study (such as nonrepresentative sample, few events per predictor, missing data).
Interpretation	19a	V	For validation, discuss the results with reference to performance in the development data, and any other validation data.
	19b	D;V	Give an overall interpretation of the results, considering objectives, limitations, results from similar studies, and other relevant evidence.
Implications	20	D;V	Discuss the potential clinical use of the model and implications for future research.
**Other information**			
Supplementary information	21	D;V	Provide information about the availability of supplementary resources, such as study protocol, Web calculator, and data sets.
Funding	22	D;V	Give the source of funding and the role of the funders for the present study.

(Continued table)

Item	R1	R2	R3	R4	R5	R6	R7	R8	R9	R10	R11	R12	R13	R14
1	×	×	√	√	√	√	√	√	√	√	×	√	√	√
2	√	√	√	√	√	√	√	√	√	×	√	√	√	√
3a	√	√	√	√	√	√	√	√	√	√	√	√	√	√
3b	√	×	√	√	√	×	√	√	√	√	√	√	√	√
4a	√	√	√	√	√	√	√	√	√	√	√	√	√	√
4b	√	×	√	√	√	√	√	√	√	?	√	√	√	√

5a	√	√	√	√	√	√	√	√	√	√	×	√	√	√
5b	√	√	√	√	√	√	√	√	√	√	√	√	√	√
5c	×	×	×	?	×	×	×	×	×	×	×	×	×	×
6a	√	√	√	√	√	√	√	√	√	√	√	√	√	√
6b	×	×	×	×	×	×	×	×	×	×	×	×	×	×
7a	√	√	√	√	√	√	√	√	√	√	√	√	√	√
7b	×	√	×	×	×	×	×	×	×	×	×	×	×	×
8	×	×	×	×	×	×	×	×	×	×	×	×	×	√
9	×	√	√	√	√	√	√	√	√	√	√	√	√	√
10a	√	√	√	√	√	√	√	√	√	√	√	√	√	√
10b	×	√	√	√	√	√	?	√	√	√	√	√	√	√
10c	×	×	×	?	?	?	?	?	×	?	×	×	?	?
10d	√	√	√	√	√	√	√	√	√	√	√	√	√	√
10e	×	×	×	×	×	×	×	×	×	×	×	×	×	×
11	√	×	×	×	√	×	√	×	×	×	×	×	√	×

12	×	×	×	√	√	×	×	√	×	√	×	×	√	√
13a	√	√	√	√	√	√	√	√	√	√	√	√	√	√
13b	×	√	√	√	√	√	×	√	√	×	×	×	×	√
13c	×	×	×	√	√	×	?	√	×	√	×	×	√	√
14a	√	√	√	√	√	√	?	√	×	√	√	√	×	√
14b	×	×	×	×	×	×	×	×	×	×	×	√	√	×
15a	×	×	×	×	√	×	√	×	×	×	√	×	×	×
15b	√	×	×	√	√	√	√	√	√	×	√	×	√	×
16	×	×	√	√	√	×	√	×	√	×	×	×	√	√
17	×	×	×	?	×	×	×	×	×	×	×	×	×	×
18	√	√	√	√	√	√	√	√	√	√	√	√	√	√
19a	×	×	×	√	√	√	√	√	√	√	×	×	√	√
19b	√	√	√	√	√	√	√	√	√	√	√	√	√	√
20	√	√	√	√	√	√	√	√	√	√	√	√	√	√
21	√	√	√	√	√	√	√	√	√	√	√	√	√	√

22	√	√	√	√	√	√	√	√	√	√	√	√	√	√

Items relevant only to the development of a prediction model are denoted by D, items relating solely to a validation of a prediction model are denoted by V, and items relating to both are denoted D; V. We recommend using the TRIPOD Checklist in conjunction with the TRIPOD Explanation and Elaboration document. "√" means "Yes", "×" means "No". The R (R1-R14) in the table indicates the research included in this review, and its order corresponds to the citation order of reference in the Supplementary Materials file and Table S2-S3.

Supplementary Material Reference

1. Peng LN, Chen LK, Chen TJ, et al. Comparisons between hypothesis- and data-driven approaches fo r multimorbidity frailty index: a machine learning approach. J Med Internet Res. 2020;22(6):e16213. d oi:10.2196/16213
2. Gomez-Cabrero D, Walter S, Abugessaisa I, et al. A robust machine learning framework to identify signatures for frailty: a nested case-control study in four aging European cohorts. GeroScience. 2021; 43(3):1317–1329. doi:10.1007/s11357-021-00334-0
3. Wu Y, Jia M, Xiang C, Fang Y. Latent trajectories of frailty and risk prediction models among geria tric community dwellers: an interpretable machine learning perspective. BMC Geriatr. 2022;22:900. do i:10.1186/s12877-022-03576-5
4. Liu Q, Yang L, Shi Z, et al. Development and validation of a preliminary clinical support system fo r measuring the probability of incident 2-year (pre)frailty among community-dwelling older adults: a prospective cohort study. Int J Med Inform. 2023;177:105138. doi:10.1016/j.ijmedinf.2023.105138
5. Liu Q, Si H, Li Y, Zhou W, Yu J, Bian Y, et al. Development and validation of a risk scoring tool for predicting incident reversible cognitive frailty among community-dwelling older adults. Geriatr G erontol Int. 2024;24(9):874–882. doi:10.1111/ggi.14942
6. Zhang W, Wang J, Xie F, Wang X, Dong S, Luo N, Li F, Li Y. Development and validation of mac hine learning models to predict frailty risk for elderly. J Adv Nurs. 2024;80(12):5064–5075. doi:10.111 1/jan.16192
7. Liu Q, Si H, Li Y, Zhou W, Yu J, Bian Y, et al. Development and validation of prediction models f or incident reversible cognitive frailty based on social-ecological predictors using generalized linear mixed model and machine learning algorithms: a prospective cohort study. J Appl Gerontol. 2025;44 (2):255–266. doi:10.1177/07334648241270052
8. Huang L, Chen H, Liang Z, et al. Enhancing the convenience of frailty index assessment for elderly Chinese people with machine learning methods. Sci Rep. 2024;14:23227. doi:10.1038/s41598-024-74194-x
9. Du C, Zhang Z, Liu B, Cao Z, Jiang N, Zhang Z. Explainable machine learning model for pre-frailt y risk assessment in community-dwelling older adults. Health Care Sci. 2024;3:426–437. doi:10.1002/hc s2.120
10. Hughes CML, Zhang Y, Pourhossein A, Jurasova T. A comparative analysis of binary and multi-clas s classification machine learning algorithms to detect current frailty status using the English Longitu dinal Study of Ageing (ELSA). Front Aging. 2025;6:1501168. doi:10.3389/fragi.2025.1501168
11. Park C, Kim N, Won CW, Kim M. Predicting cognitive frailty in community-dwelling older adults: a machine learning approach based on multidomain risk factors. Sci Rep. 2025;15:18369. doi:10.1038/s 41598-025-00844-3
12. Qi L, Liu J, Song X, et al. Determinants and risk prediction models for frailty among community-liv ing older adults in eastern China. Front Public Health. 2025;13:1518472. doi:10.3389/fpubh.2025.151847 2
13. Dong Y, Wang Q, Zhang K, et al. Development and validation of a prediction model of frailty risk in community-dwelling older adults: from a national longitudinal survey. Public Health. 2025;240:63–70. doi:10.1016/j.puhe.2024.12.055
14. Isaradech N, Sirikul W, Buawangpong N, Siviroj P, Kitro A. Machine learning models for frailty cla ssification of older adults in Northern Thailand: model development and validation study. JMIR Agi ng. 2025;8:e62942. doi:10.2196/62942

Table S6. Supplementary database search of IEEE Xplore and ACM Digital Library (May 2026): PRISMA stage summary.

Stage	IEEE Xplore	ACM Digital Library	Total
Records identified	18	24	42
Records removed before title/abstract screening	0	0	0
Records screened at title/abstract stage	18	24	42
Excluded at title/abstract screening	12	23	35
Retained for full-text assessment	6	1	7
Excluded at full-text eligibility	6	1	7
New studies eligible for inclusion	0	0	0

Table S7. Per-record exclusion of seven full-text candidates identified through the supplementary IEEE Xplore and ACM Digital Library search (May 2026).

#	Author, Year	Database	Sample size / Age	Reason for exclusion
1	Olugbenga et al., 2025	IEEE Xplore	n = 2,173 / mixed (AGELESS ≥60, MELoR ≥55)	Age criterion not met: the combined cohort may include participants younger than 60 years because MELoR included adults aged ≥55, and the article did not provide a separate ≥60-only analysis.
2	Amjad et al., 2025	IEEE Xplore	n = 682 / ≥65 yr	Sample size below 1,000 (prespecified PROSPERO criterion).
3	Eskandari et al., 2022	IEEE Xplore	n = 88 / ≥65 yr	Sample size below 1,000 (prespecified PROSPERO criterion).
4	Minici et al., 2022	IEEE Xplore	n = 34 / ≥65 yr	Sample size below 1,000 (prespecified PROSPERO criterion).

5	Jung et al., 2021	IEEE Xplore	Same record as in main search	Search-record duplicate: this record was retrieved through the supplementary IEEE Xplore search but corresponded to primary-search record R23, which had already been excluded at full-text assessment because of sample size below 1,000 (n = 74). Therefore, it was not a new eligible study.
6	Bertini et al., 2018	IEEE Xplore	n = 95,368 / ≥65 yr	Construct validity: frailty was operationalized as a surrogate outcome (1-year emergency hospitalization or all-cause mortality), not via a validated frailty assessment instrument.
7	Ozaki et al., 2024	ACM Digital Library	n = 28 / ≥65 yr	Sample size below 1,000 (prespecified PROSPERO criterion).

Note. The supplementary database search retrieved 42 records (IEEE Xplore n=18; ACM Digital Library n=24). No records were removed before title/abstract screening. Two search-record duplicates of primary-search records were identified during screening rather than removed at upfront deduplication: one was excluded at the title/abstract stage because it was a systematic review already screened in the primary search (Yang et al., 2025), and one was excluded at the full-text eligibility stage because it was the same paper as primary-search record R23 (Jung et al., 2021), which had already been excluded for sample size below 1,000 (n = 74). Thirty-five records were excluded at the title/abstract screening stage, and seven were retained for full-text assessment. All seven were excluded at the eligibility stage as detailed in Table S7. The supplementary search therefore yielded no additional eligible studies, and the original 14-study inclusion was retained. The complete list of retrieved records and the per-record screening decisions are openly available in the GitHub repository: https://github.com/delic1758/ml-frailty-systematic-review-materials.

PRISMA 2020 Checklist

Manuscript: Machine Learning–Based Frailty Prediction and Classification in Community-Dwelling Older Adults: A Systematic Review of Validation, Explainability, and Implementation Readiness

Manuscript ID: healthcare-4324907

Section and Topic	Item #	Checklist item	Location in manuscript
**TITLE**			
Title	1	Identify the report as a systematic review.	Title (p. 1): "A Systematic Review of Validation, Explainability, and Implementation Readiness".
**ABSTRACT**			
Abstract	2	See the PRISMA 2020 for Abstracts checklist.	Abstract (§ Abstract).
**INTRODUCTION**			
Rationale	3	Describe the rationale for the review in the context of existing knowledge.	§1 (Introduction).
Objectives	4	Provide an explicit statement of the objective(s) or question(s) the review addresses.	§1 (Introduction), end of section.
**METHODS**			
Eligibility criteria	5	Specify the inclusion and exclusion criteria for the review and how studies were grouped for the syntheses.	§2.3 (Eligibility Criteria).
Information sources	6	Specify all databases, registers, websites, organisations, reference lists and other sources searched or consulted to identify studies. Specify the date when each source was last searched or consulted.	§2.2 (Information Sources and Search Strategy); Supplementary Table S1.
Search strategy	7	Present the full search strategies for all databases, registers and websites, including any filters and limits used.	§2.2 (main search 4 Jul 2025; supplementary IEEE/ACM search May 2026); Supplementary Table S1.

Selection process	8	Specify the methods used to decide whether a study met the inclusion criteria of the review, including how many reviewers screened each record and each report retrieved, whether they worked independently, and if applicable, details of automation tools used in the process.	§2.3 (Selection Process): single primary reviewer with independent verification by second reviewer (S.K. → J.-H.P.); EndNote duplicate removal.
Data collection process	9	Specify the methods used to collect data from reports, including how many reviewers collected data from each report, whether they worked independently, any processes for obtaining or confirming data from study investigators, and if applicable, details of automation tools used in the process.	§2.4 (Data Extraction and Synthesis).
Data items	10a	List and define all outcomes for which data were sought. Specify whether all results that were compatible with each outcome domain in each study were sought (e.g., for all measures, time points, analyses), and if not, the methods used to decide which results to collect.	§2.4; Tables 3–5; Supplementary Tables S2–S3.
	10b	List and define all other variables for which data were sought (e.g., participant and intervention characteristics, funding	§2.4; Supplementary Table S4 (missing-data handling).

		sources). Describe any assumptions made about any missing or unclear information.	
Study risk of bias assessment	11	Specify the methods used to assess risk of bias in the included studies, including details of the tool(s) used, how many reviewers assessed each study and whether they worked independently, and if applicable, details of automation tools used in the process.	§2.4 (PROBAST); §3.10 and Table 7; Supplementary Table S5 (TRIPOD).
Effect measures	12	Specify for each outcome the effect measure(s) (e.g., risk ratio, mean difference) used in the synthesis or presentation of results.	§2.4 (AUROC, AUPRC, calibration, Brier score, DCA); Table 5; Figure 4.
Synthesis methods	13a	Describe the processes used to decide which studies were eligible for each synthesis (e.g., tabulating the study intervention characteristics and comparing against the planned groups for each synthesis).	§2.4 (Narrative Synthesis); Tables 1–7.
	13b	Describe any methods required to prepare the data for presentation or synthesis, such as handling of missing summary statistics or data conversions.	§2.4; Supplementary Table S4.
	13c	Describe any methods used to tabulate or visually display results of individual studies and syntheses.	§2.4; Figures 1–4; Tables 1–7.

	13d	Describe any methods used to synthesise results and provide a rationale for the choice(s). If meta-analysis was performed, describe the model(s), method(s) to identify the presence and extent of statistical heterogeneity, and software package(s) used.	§2.4 (Narrative synthesis; no meta-analysis owing to heterogeneity in frailty operationalizations, predictor sets, validation strategies, and outcome formulations).
	13e	Describe any methods used to explore possible causes of heterogeneity among study results (e.g., subgroup analysis, meta-regression).	§4.1–§4.2 (qualitative exploration of heterogeneity by task type, validation type, frailty operationalization).
	13f	Describe any sensitivity analyses conducted to assess robustness of the synthesised results.	§4.5 (Strengths and Limitations); supplementary search as de facto sensitivity check.
Reporting bias assessment	14	Describe any methods used to assess risk of bias due to missing results in a synthesis (arising from reporting biases).	§2.4; §4.5 (publication bias and database-coverage discussion).
Certainty assessment	15	Describe any methods used to assess certainty (or confidence) in the body of evidence for an outcome.	§3.10 (PROBAST, Table 7); §4.4 (RE-AIM / TRL synthesis, Table 6); §4.5 (Strengths and Limitations).
**RESULTS**			
Study selection	16a	Describe the results of the search and selection process, from the number of records identified in the search to the number of studies included in the review, ideally using a flow diagram.	§3.1; Figure 1 (revised PRISMA 2020 dual-track flow diagram including IEEE/ACM supplementary search).
	16b	Cite studies that might appear to meet the inclusion criteria, but which	§2.3 (¶53); §4.5 (Bertini construct-validity exclusion); Supplementary Tables S6–S7.

		were excluded, and explain why they were excluded.	
Study characteristics	17	Cite each included study and present its characteristics.	Tables 1–4; §3.2–§3.4; Supplementary Tables S2–S3.
Risk of bias in studies	18	Present assessments of risk of bias for each included study.	§3.10; Table 7; Supplementary Table S5.
Results of individual studies	19	For all outcomes, present, for each study: (a) summary statistics for each group (where appropriate) and (b) an effect estimate and its precision (e.g., confidence/credible interval), ideally using structured tables or plots.	Table 5; Figure 4; Supplementary Table S2.
Results of syntheses	20a	For each synthesis, briefly summarise the characteristics and risk of bias among contributing studies.	§3.2–§3.10; Tables 1–7.
	20b	Present results of all statistical syntheses conducted. If meta-analysis was done, present for each the summary estimate and its precision (e.g., confidence/credible interval) and measures of statistical heterogeneity. If comparing groups, describe the direction of the effect.	§3.5 (Performance); Figure 4 (descriptive, not meta-analytic — pooled estimates not computed owing to heterogeneity).
	20c	Present results of all investigations of possible causes of heterogeneity among study results.	§4.1–§4.2 (heterogeneity sources discussed qualitatively).
	20d	Present results of all sensitivity analyses conducted to assess the	§4.5 (Strengths and Limitations); supplementary IEEE/ACM search reported transparently.

		robustness of the synthesised results.	
Reporting biases	21	Present assessments of risk of bias due to missing results (arising from reporting biases) for each synthesis assessed.	§4.5 (¶130–131).
Certainty of evidence	22	Present assessments of certainty (or confidence) in the body of evidence for each outcome assessed.	§3.10 (PROBAST, Table 7); §4.4 (Table 6 RE-AIM / TRL).
**DISCUSSION**			
Discussion	23a	Provide a general interpretation of the results in the context of other evidence.	§4.1–§4.6 (Discussion subsections).
	23b	Discuss any limitations of the evidence included in the review.	§4.5 (Strengths and Limitations).
	23c	Discuss any limitations of the review processes used.	§4.5 (Strengths and Limitations).
	23d	Discuss implications of the results for practice, policy, and future research.	§4.6 (Implications for Practice and Future Research); §5 (Conclusions).
**OTHER INFORMATION**			
Registration and protocol	24a	Provide registration information for the review, including the register name and registration number, or state that the review was not registered.	§2.1 (PROSPERO CRD420251081555).
	24b	Indicate where the review protocol can be accessed, or state that a protocol was not prepared.	PROSPERO record (publicly accessible).
	24c	Describe and explain any amendments to information provided at	§2.2 (May 2026 supplementary IEEE/ACM search added in response to peer review; eligibility

		registration or in the protocol.	criteria refined to require validated phenotypic frailty assessment, reported transparently in §2.3 and §4.5).
Support	25	Describe sources of financial or non-financial support for the review, and the role of the funders or sponsors in the review.	Funding statement.
Competing interests	26	Declare any competing interests of review authors.	Conflicts of Interest statement.
Availability of data, code, and other materials	27	Report which of the following are publicly available and where they can be found: template data collection forms; data extracted from included studies; data used for all analyses; analytic code; any other materials used in the review.	Data Availability Statement: https://github.com/delic1758/ml-frailty-systematic-review-materials (data, code, screening records, PROBAST/TRIPOD assessments, figure source data).

Adapted from: Page MJ, McKenzie JE, Bossuyt PM, Boutron I, Hoffmann TC, Mulrow CD, et al. The PRISMA 2020 statement: an updated guideline for reporting systematic reviews. BMJ 2021;372:n71. doi:10.1136/bmj.n71. For more information, visit: http://www.prisma-statement.org/
